# Policy text analysis of China’s smart pharmaceutical regulation under a policy tool–policy stage framework

**DOI:** 10.3389/fpubh.2026.1848335

**Published:** 2026-06-17

**Authors:** Zhaodi Yu, Zhenxiang Xu, Jiangang Qi

**Affiliations:** 1 Law School, Zhongnan University of Economics and Law, Wuhan, Hubei, China; 2 Research Center of Intellectual Property, Zhongnan University of Economics and Law, Wuhan, Hubei, China; 3 Beijing Guantao (Wuhan) Law Firm, Wuhan, China

**Keywords:** content analysis, NATO framework, policy instruments, policy stages, smart pharmaceutical regulation

## Abstract

Smart pharmaceutical regulation has emerged as a key approach to enhancing regulatory capacity and ensuring drug safety in the context of digital government. However, existing studies have largely focused on technological applications or descriptive policy analyses, with limited attention to the structural characteristics and evolution of policy instruments. To address this gap, this study applies a two-dimensional analytical framework that integrates policy instruments and policy development stages to examine China’s smart pharmaceutical regulation policies. Drawing on a dataset of 90 policy documents issued between 2007 and 2026, the study employs a content-analysis approach, using the Nodality, Authority, Treasure, and Organization (NATO) framework to classify policy instruments and NVivo software to support systematic coding. The findings showed that nodality-based instruments constitute the largest share of the policy texts, reflecting a strong policy emphasis on data-driven governance, information sharing, and digital regulatory capacities. Treasure-based instruments primarily focus on digital infrastructure and technical-capacity development, while authority-based instruments continue to play an important role alongside emerging digital regulatory tools. Conversely, organization-based instruments receive comparatively less emphasis in the policy text, particularly regarding institutional coordination and stakeholder co-governance. From a temporal perspective, the policy texts reflect a shift from informationization-oriented regulation toward digital integration and, more recently, toward lifecycle-based and intelligence-oriented regulatory arrangements. Nevertheless, structural imbalances in the distribution of policy instruments remain evident within the policy corpus. The findings suggest the importance of strengthening interagency coordination, stakeholder participation, and the integration of technological and institutional governance mechanisms in future policy design. This study contributes to the literature by providing a systematic policy text analysis of smart pharmaceutical regulation and offering insights into the evolving structure of regulatory governance in the digital age.

## 1 Introduction

Safe, effective, and accessible medicines are indispensable to human survival and development. Access to essential, high-quality medicines is widely recognized as a fundamental component of the right to health (1). Following the 1937 sulfanilamide disaster, the United States Congress enacted the 1938 *Federal Food, Drug, and Cosmetic Act* (FDCA), which established the Food and Drug Administration (FDA). Through mechanisms such as the new drug approval process, the FDA was empowered to regulate the production and marketing of pharmaceuticals (2, 3). Since then, modern systems of pharmaceutical regulation have gradually spread worldwide, with jurisdictions establishing national regulatory authorities for medicines (4–6).

As a core domain of government regulation, pharmaceutical regulation aims to safeguard public health by ensuring the safety, efficacy, and quality of medicines throughout their lifecycle (7). More specifically, it encompasses a combination of legal, administrative, and technical measures through which governments oversee drug development, production, distribution, and use, as well as the accuracy and reliability of related information (1).

Traditional pharmaceutical regulation is primarily characterized by a bureaucratic and compliance-oriented model centered on ex ante authorization and ex post enforcement. Regulatory interventions extend across multiple stages of the pharmaceutical lifecycle, including research and development, production, and market entry (8). However, traditional pharmaceutical regulation faces several challenges, including cross-agency collaboration and information integration, the limited institutionalization of stakeholder co-governance and public participation, and delays in risk identification due to underreporting in pharmacovigilance systems (9, 10).

In response to these limitations, the rapid development and application of digital technologies, including cloud computing, big data, blockchain, and artificial intelligence, are reshaping pharmaceutical regulation. Cloud-based platforms support regulatory submissions, data sharing, and collaboration between regulators and industry stakeholders (11). Big data enables validation of claims made during the approval process by facilitating large-scale analysis of patient-level data to assess the safety, effectiveness, and real-world performance of medical products (12). Blockchain enhances drug traceability and supply chain transparency through decentralized data sharing, reducing the risks of data manipulation and information asymmetry (13). Meanwhile, artificial intelligence automates key regulatory processes, including data extraction, dossier preparation, and compliance management, improving efficiency and enabling more informed regulatory decision-making (14).

In China’s regulatory context, the application of these emerging technologies to pharmaceutical regulation is collectively referred to by regulatory authorities as “Smart Pharmaceutical Regulation.” This model represents a key direction in the transformation of China’s pharmaceutical regulatory system and serves as an important means of enhancing regulatory capacity, building a more scientific and efficient regulatory framework, and ensuring the safety of public medication use (15). In recent years, a series of key policy documents in the field of pharmaceutical regulation have consistently emphasized the promotion of smart pharmaceutical regulation. For example, the National Medical Products Administration (NMPA) issued the *Action Plan for Accelerating the Development of Smart Pharmaceutical Regulation* in 2019, which emphasizes integrating regulatory practices with digital technologies such as cloud computing, big data, and the “Internet+” initiative to innovate regulatory approaches and support reform and development (16). Furthermore, the *14th Five-Year Plan for National Drug Safety and High-Quality Development*, jointly released by the NMPA and seven other departments in 2021, highlights key priorities for smart pharmaceutical regulation, including the establishment of an information-based drug traceability system, the promotion of lifecycle digital management of pharmaceuticals, the development of standardized regulatory information systems, and the enhancement of “Internet+ drug regulation” services (17).

However, existing studies primarily focus on the application of digital technologies in pharmaceutical regulation (18), descriptive analyses and policy interpretations of smart pharmaceutical regulation (19), and single-dimensional analyses of specific areas within it (20). Empirical research on the use of policy instruments and their structural characteristics in smart pharmaceutical regulation remains limited, and few studies have systematically examined how regulatory instruments evolve across different stages of policy development. In this context, as a central means of government regulation, policy instruments provide a crucial analytical lens for understanding the development and improvement of smart pharmaceutical regulation. In light of these gaps, this study applies the Nodality, Authority, Treasure, and Organization (NATO) framework of policy instruments to analyze policy texts on smart pharmaceutical regulation in China from two dimensions, namely policy instruments and policy stages, and proposes corresponding policy recommendations based on the structural issues identified in current instrument use.

This study offers three key contributions: First, it moves beyond a predominantly technology-oriented discussion of smart pharmaceutical regulation by examining the policy-instrument structure as reflected in official policy texts. This perspective clarifies how smart pharmaceutical regulation is represented in policy design. Second, it adapts the NATO policy instrument framework to smart pharmaceutical regulation by developing second-level sub-nodes that reflect digital governance, digital administrative law, and China’s pharmaceutical regulatory context, and uses this framework to describe the distribution and temporal evolution of policy instruments across different development stages. Third, by identifying the relative underdevelopment of organization-based instruments, the study further proposes institutional pathways for strengthening interagency coordination, stakeholder participation, and lifecycle-oriented regulatory governance. In doing so, it provides a policy-oriented basis for improving the institutional design of smart pharmaceutical regulation in China.

## 2 Materials and methods

### 2.1 Sources of materials

Policy documents were collected from multiple authoritative legal and policy databases to construct a comprehensive corpus of China’s smart pharmaceutical regulation policies. The corpus covered policy documents issued between January 1, 2005, and March 1, 2026, with March 1, 2026, set as the final retrieval cutoff date. Because this study examines policy design rather than implementation outcomes, only officially published policy documents issued by central state organs and provincial-level governments or their competent departments were included.

The search covered four types of sources. First, the Peking University Legal Information Network (PKULaw) was used as the primary legal and policy database for retrieving laws, regulations, departmental rules, normative documents, plans, notices, and opinions related to pharmaceutical regulation. Smart pharmaceutical regulation is a specialized and emerging area within China’s broader pharmaceutical regulatory system. During the retrieval process, it was found that many relevant policy texts were not issued as formal legislation or administrative regulations in the narrow sense, but rather as policy documents and administrative normative documents, such as action plans, implementation opinions, notices, and development plans issued by administrative authorities. Although these documents do not necessarily constitute formal legislation, they still possess important normative and governance functions within China’s administrative system. Therefore, to ensure the comprehensiveness and representativeness of the policy corpus, additional sources beyond PKULaw were incorporated into the retrieval process. Second, the policy document database issued by the State Council was searched to identify central-level policy documents issued by the State Council and by national ministries or commissions. Third, official websites of provincial governments and provincial medical products administrations were searched to identify provincial-level policies. Fourth, the China National Knowledge Infrastructure (CNKI) was used as a supplementary source to cross-check policy titles, identify policy interpretations, and avoid omissions in policy retrieval.

The original Chinese search terms, with English translations provided for reference, included: “药品智慧监管” (“smart pharmaceutical regulation”), “智慧药品监管” (“pharmaceutical smart regulation”), “药品监管信息化” (“informatization of pharmaceutical regulation”), “药品监管数字化” (“digitalization of pharmaceutical regulation”). Where database functions allowed, the search was conducted in the title, full text, and keyword fields. For official government websites, the same Chinese search terms were used through website-level search functions and, where necessary, through manual browsing of policy document columns.

The preliminary retrieval conducted primarily through PKULaw yielded 109 potentially relevant policy documents. To improve retrieval comprehensiveness and reduce the risk of omissions, supplementary searches and cross-checking were subsequently conducted using the policy document database issued by the State Council, official websites of provincial governments and provincial medical products administrations, and CNKI. Through this supplementary retrieval process, an additional 6 potentially relevant policy documents were identified, bringing the preliminary corpus of 115 policy documents. The resulting preliminary corpus was then screened according to the following inclusion and exclusion criteria.

The inclusion criteria were as follows:

1. The document was issued by a central state organ, national ministry or commission, provincial-level government, or provincial-level competent department.
2. The document was officially published and publicly accessible.
3. The document was substantively related to smart pharmaceutical regulation, pharmaceutical regulatory informatization, digital pharmaceutical supervision, pharmaceutical traceability, or regulatory capacity building in the pharmaceutical field.
4. The document was issued between January 1, 2005, and March 1, 2026.

The exclusion criteria were as follows:

1. Documents issued by municipal, county, township, or lower-level authorities were excluded to maintain a central/provincial comparative structure.
2. Documents that only mentioned smart pharmaceutical regulation or related terms without substantive regulatory content were excluded.
3. Duplicate documents retrieved from multiple sources were excluded.
4. News reports, policy interpretations, academic articles, and non-normative publicity materials were excluded.

**Figure 1:**
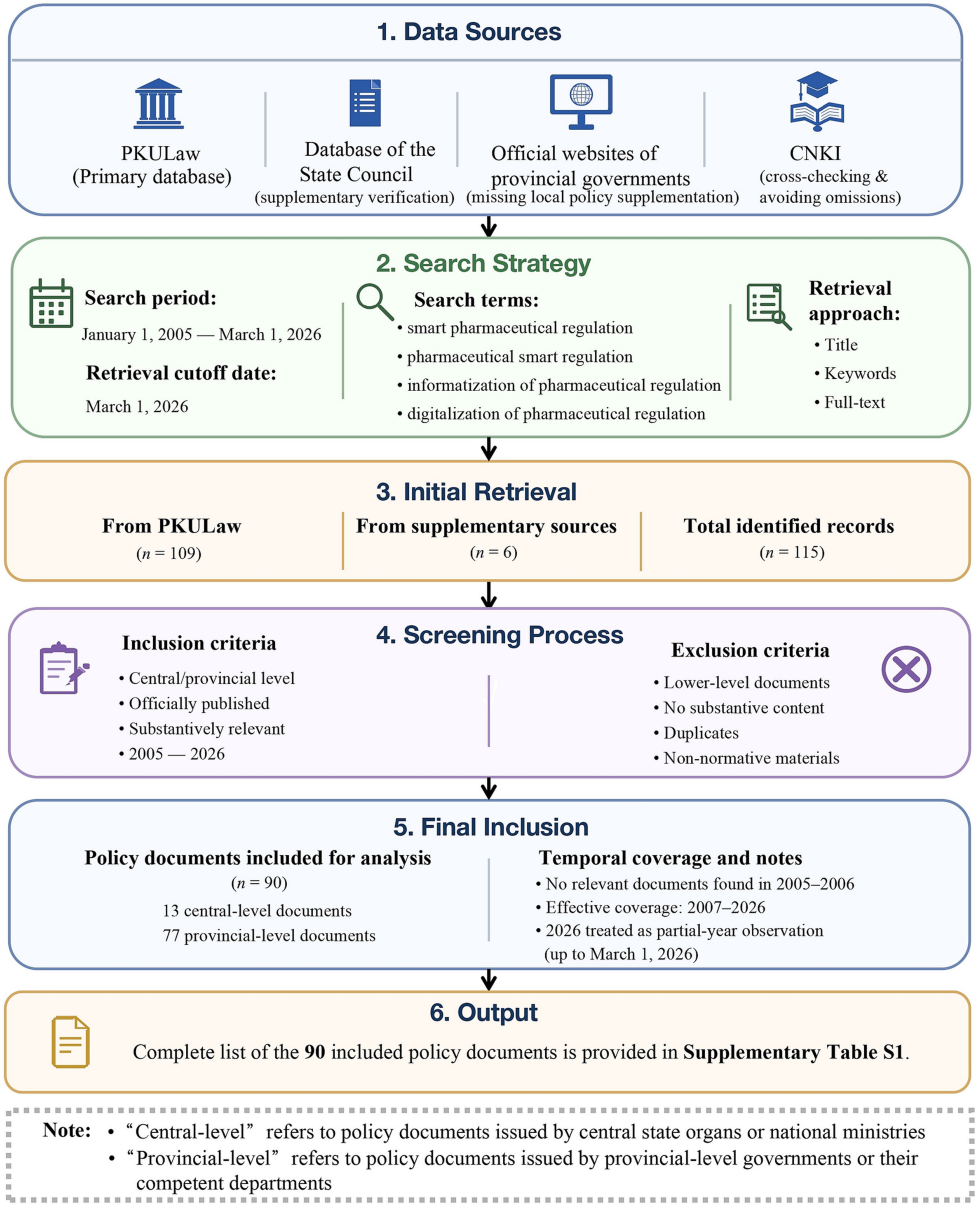
Retrieval and screening process of policy documents.

After screening, 90 policy documents issued between 2005 and 2026 were retained for analysis, including 13 central-level and 77 provincial-level documents. Although the retrieval scope covered the period from 2005 to 2026, no substantively relevant policy documents were identified for 2005 and 2006. Therefore, the final policy corpus spans 2007 to 2026. The year 2026 was treated as a partial-year observation, with documents included only up to the retrieval cutoff date of March 1, 2026. Thus, annual counts for 2026 should not be directly compared with complete annual counts for previous years. The overall retrieval and screening process is illustrated in Figure 1. The complete list of the 90 included policy documents is provided in Supplementary Table S1.

### 2.2 Analysis framework

**Figure 2:**
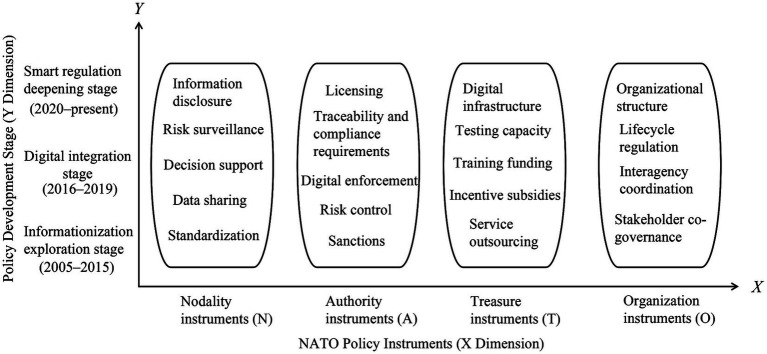
A two-dimensional analytical framework of smart pharmaceutical regulation based on policy instruments and policy development stages.

This study constructs a two-dimensional analytical framework that integrates policy instruments and policy development stages. The X dimension represents policy instruments, while the Y dimension captures the temporal evolution of policy development stages, as illustrated in Figure 2.

#### 2.2.1 Dimension X: policy instrument classification based on the NATO framework

Policy instruments refer to the tools and techniques used by governments to achieve policy objectives and influence the behavior of relevant actors (21). Various frameworks have been proposed to classify policy instruments in public policy research (22–24). Among them, the NATO framework proposed by Hood classifies policy instruments into four categories according to the key governance resources available to governments: NATO (25, 26). Due to its clear analytical logic and wide applicability, the NATO framework has been widely adopted in policy instrument studies (27–29).

Given that smart pharmaceutical regulation involves information management, regulatory authority, resource allocation, and organizational coordination, the NATO framework is an appropriate analytical framework for this study. However, the original NATO framework was primarily developed in the context of traditional public administration and may not fully capture the characteristics of digital governance (30), including platform-based regulation, data sharing, algorithmic decision-making, lifecycle governance, and networked coordination (31–33).

To address these limitations, this study retained the NATO framework as the first-level analytical structure while adapting the construction of second-level sub-nodes to reflect the characteristics of smart pharmaceutical regulation and the broader perspectives of digital governance and digital administrative law (34, 35). In expanding the analytical scope of the policy-instrument framework toward digital governance, the study also incorporated core concerns derived from administrative law and regulatory governance theories, including risk regulation, administrative discretion and decision-making, standardization, administrative compliance, administrative enforcement, administrative sanctions, administrative incentives, and stakeholder participation. This adaptation enabled the framework to better capture the procedural, coordinative, and technology-mediated dimensions of smart pharmaceutical regulation within the broader resource-oriented structure of the NATO framework. The detailed classification and descriptions of the primary nodes and sub-nodes are presented in Table 1.

**Table 1:** Classification of policy instruments based on the NATO framework.

Policy instrument category	Definition	Sub-nodes	Description
Nodality-based instruments	Policy instruments that rely on information resources and knowledge dissemination to influence the behavior of policy actors. Governments use data collection, information sharing, and communication mechanisms to guide regulatory activities.	Information disclosure	Publication of inspection results, risk alerts, regulatory notices, and public education on safe medication.
		Risk surveillance	Continuous monitoring of regulated entities, products, or behaviors to identify potential risks and provide early warning signals.
		Decision support	Use of data analytics and intelligent systems to support regulatory decision-making, including risk modeling and classification-based supervision.
		Data sharing	Integration and exchange of regulatory data across platforms and agencies to support coordinated supervision.
		Standardization	Development of data standards, regulatory guidelines, and technical norms to ensure consistency in regulatory practices.
Authority-based instruments	Policy instruments grounded in legal authority and regulatory mandates that require regulated actors to comply with formal rules and to be subject to enforcement mechanisms.	Licensing	Online licensing, registration, and filing procedures for pharmaceutical production and distribution activities.
		Traceability and compliance requirements	Mandatory compliance obligations such as GMP/GSP standards, reporting duties, and traceability requirements in smart regulation.
		Digital enforcement	Application of digital technologies in regulatory inspections and enforcement activities.
		Risk control	Regulatory mechanisms are designed to manage risks, including ADR monitoring systems and product recall procedures.
		Sanctions	Administrative penalties are imposed on non-compliant actors, including fines, license suspension, and other enforcement measures.
Treasure-based instruments	Policy instruments that utilize financial resources to support regulatory capacity building and encourage compliance with policy objectives.	Digital infrastructure	Government investment in digital platforms, regulatory information systems, and data centers.
		Testing capacity	Financial support for laboratories, testing equipment, and inspection facilities.
		Training funding	Funding programs aimed at improving the professional capacity of regulatory personnel.
		Incentive subsidies	Financial incentives, pilot project funding, and subsidies encourage regulatory innovation and digital transformation.
		Service outsourcing	Government procurement of services from third-party institutions, including inspection services and platform operation.
Organization-based instruments	Policy instruments that rely on organizational arrangements, institutional coordination, and administrative capacity to achieve policy objectives.	Organizational structure	Establishment of regulatory institutions, dedicated teams, and clearly defined responsibilities.
		Lifecycle regulation	Regulatory mechanisms that cover the entire lifecycle of pharmaceutical products from production to post-market supervision.
		Interagency coordination	Collaboration among multiple regulatory authorities and government departments.
		Stakeholder co-governance	Participation of experts, technical institutions, social organizations, and the public in collaborative regulatory governance.

#### 2.2.2 Dimension Y: policy development stages of smart pharmaceutical regulation

The Y dimension represents the temporal evolution of smart pharmaceutical regulation policies in China. The stage division was informed by the literature on the evolution of digital and smart government. Government digitalization tends to progress from basic digitization and technology use within government toward organizational transformation, stakeholder engagement, and context-specific, policy-driven digital governance (36). Smart government studies further indicate that later stages of digital transformation increasingly rely on emerging technologies, such as big data, artificial intelligence, cloud computing, blockchain, and the Internet of Things, to support more integrated, intelligent, and data-driven public governance (37). Since pharmaceutical safety regulation is a sector-specific public regulatory function through which digital government transformation is implemented, this study used the degree of digital technology application, the transformation of regulatory architecture, and the evolution of governance functions, as reflected in policy texts, as key criteria for identifying the development stages of smart pharmaceutical regulation. The policy development process was divided into three analytically constructed stages. These stages were defined before the formal matrix coding and quantitative comparison. The criteria and representative policy text evidence used to identify the three stages are summarized in Table 2.

**Table 2:** Criteria for identifying policy development stages of smart pharmaceutical regulation.

Stage	Period	Main criteria for stage identification	Representative policy document
Informationization exploration stage	2005–2015	Initial use of information technology in pharmaceutical regulation; construction of electronic supervision systems and basic regulatory databases; digital tools mainly used to assist routine administrative management	Drug electronic regulation work plan (2011–2015)
Digital integration stage	2016–2019	Shift from fragmented information systems to data integration, platform-based supervision, and pharmaceutical traceability; growing emphasis on “Internet Plus Regulation” and regulatory data sharing	13th five-year national drug safety plan
Smart regulation deepening stage	2020–present	Expansion from platform integration to data-driven, risk-based, lifecycle-oriented, and intelligent regulation; increasing use of big data, AI, cloud computing, blockchain, intelligent warning, and digital enforcement	Action plan for accelerating the development of smart pharmaceutical regulation

The first stage is the informationization exploration stage (2005–2015). This stage corresponds to the early e-government logic of digitizing administrative information and building basic information systems. In policy texts, the main concern was not yet the integration of smart governance, but the establishment of an electronic supervision infrastructure. The *2011–2015 Drug Electronic Regulation Work Plan* is representative of this stage, as it emphasized the construction of the pharmaceutical electronic supervision information platform, electronic supervision database, data center, backup center, information security system, and electronic supervision service system. These policy contents indicate that digital technology was primarily used for information collection, storage, and transmission, as well as for basic electronic supervision, whereas cross-departmental, cross-level, full-lifecycle data integration and intelligent decision-support mechanisms had not yet been established.

The second stage is the digital integration stage (2016–2019). The boundary between 2015 and 2016 reflects a shift from basic information system construction to integrated data governance, platform interconnection, traceability, and “Internet Plus Regulation.” A representative document is the *13th Five-Year National Drug Safety Plan*, which proposed building national and provincial drug safety regulatory big data centers and regulatory information platforms, improving information standards and resource management systems, and supporting regulatory functions such as administrative approval, inspection, enforcement, emergency management, risk analysis, credit management, and public services. It also emphasized establishing a full-variety, full-chain drug traceability system. These policy contents indicate that pharmaceutical regulation was moving from separate information systems toward data integration and platform-based regulatory coordination. However, artificial intelligence, algorithmic analysis, and automated decision support remained limited.

The third stage is the smart regulation deepening stage (2020–present). The 2019 NMPA *Action Plan on Accelerating Smart Pharmaceutical Regulation* served as a programmatic turning point by proposing a “large system, large platform, and big data” architecture and by emphasizing data sharing, business coordination, full-lifecycle data integration, cloud computing, big data, blockchain, and drug traceability platform construction. The boundary between 2019 and 2020 was therefore set after this agenda-setting document, because the implementation and diffusion of smart regulation became more visible from 2020 onward. In this stage, policy texts increasingly emphasized smart regulation, regulatory capacity building, risk-based supervision, full-lifecycle digital management, intelligent warning, digital enforcement, and decision support. This indicates a shift from digital integration to data-driven, risk-oriented, and lifecycle-based smart regulation.

### 2.3 Research methods

Content analysis is a systematic and replicable method that categorizes large volumes of textual data through explicit coding procedures (38). Organizing texts into analytical units enables researchers to identify patterns and themes and draw meaningful conclusions from qualitative data (39). NVivo 15 software was used to assist the analysis of policy documents in this study. As a qualitative data analysis tool, NVivo enables researchers to systematically manage, code, and visualize large volumes of textual data. It allows researchers to organize and analyze unstructured materials, identify patterns and themes through coding and querying functions, and present analytical results through visualization tools (40). These functions make NVivo particularly suitable for policy document analysis involving large datasets.

This study adopts a hybrid deductive–inductive content analysis approach (41). The classification of policy instruments was guided deductively by the NATO framework, which served as the predefined theoretical structure. The four NATO categories were used as first-level coding nodes. Based on the specific content of the policy documents, relevant thematic terms were further identified and extracted to construct second-level sub-nodes under each category. The development of second-level sub-nodes was conducted iteratively through repeated reading, open coding, and thematic refinement of the policy texts. During this process, conceptually similar policy expressions were gradually merged into broader analytical categories to improve coding consistency and analytical coherence.

In this study, the primary coding unit was a thematic segment, defined as a sentence, clause, or paragraph fragment with a relatively independent policy meaning related to policy instruments or regulatory governance. Coding was conducted based on semantic meaning rather than fixed textual length. To maintain analytical consistency and avoid artificial inflation of coding frequencies, each thematic segment was assigned to only one primary policy-instrument category. When a policy provision appeared to involve multiple governance functions, coding decisions were guided by the dominant regulatory intention and the primary governance function reflected in the text, rather than by isolated keywords or secondary policy effects. In the NVivo coding results, “coding reference points” refer to the number of coded thematic segments assigned to a particular node or sub-node, not the number of policy documents. Therefore, a single policy document could contribute multiple coding reference points across different policy-instrument categories.

Meanwhile, the policy development stages were identified through a deductive–inductive process that combined theoretical guidance from the digital government and smart government literature with iterative analysis of the policy documents’ temporal evolution and thematic characteristics. Based on this preliminary analysis, three analytically constructed stages were established before formal coding and subsequently used as case attributes in the NVivo matrix coding process. In NVivo, each policy document was treated as an individual case. A case classification titled “Policy Documents” was created to record the attributes of each policy. Within this classification, a “Stage” attribute was established to represent the policy development stages, and each policy document was assigned to one of the three stages through case attribute coding.

To improve coding transparency and reproducibility, representative coded policy segments and their coding rationales are provided in Supplementary Table S2.

### 2.4 Reliability check

To ensure the consistency and reliability of the coding process, an inter-coder reliability check was conducted using Cohen’s Kappa coefficient. Before formal coding, two researchers were trained using the coding framework presented in Table 2 and the representative examples of coding rules provided in Supplementary Table S2. The training process focused on interpreting first-level NATO categories, applying second-level sub-nodes, and handling ambiguous policy provisions. After training, the two researchers independently coded the full corpus of 90 policy documents (*n* = 90). The reliability test was conducted before discussion and consensus resolution to assess independent coding agreement rather than post-discussion agreement. The resulting Cohen’s Kappa value was 0.81, indicating a high level of agreement between the two coders (42). The reliability procedure and results are summarized in Table 3.

**Table 3:** Inter-coder reliability procedure and results.

Component	Value/description	Notes
Test type	Inter-coder reliability check	Assessed consistency of policy text coding.
Statistical metric	Cohen’s kappa coefficient (*κ*)	Measures agreement beyond chance.
Number of coders	2	Two trained independent coders.
Coding framework	Table 1 and Supplementary Table S2	Based on NATO categories and adapted second-level sub-nodes.
Sample size	90 policy documents (*n* = 90)	The full corpus was independently coded.
Calculation timing	Before discussion and consensus resolution	Kappa calculated before disagreement resolution.
Kappa value	*κ* = 0.81	Indicates a high level of agreement.
Main disagreements	Overlapping governance functions	For example, data sharing within interagency coordination.
Resolution process	Coder discussion; third researcher if needed	Based on the dominant regulatory intention.

Coding disagreements mainly arose from policy provisions involving closely related or overlapping governance functions, such as data sharing among administrative agencies within interagency coordination, digital infrastructure construction implemented through service outsourcing, and authority-based regulatory measures embedded in lifecycle regulation. These disagreements were resolved through coder discussion based on the dominant regulatory intention and primary governance function of each thematic segment. Where consensus could not be reached, a third researcher was consulted to make the final coding decision.

## 3 Results

This section presents both the selection and application of policy instruments and their distribution across the three stages of smart pharmaceutical regulation. Notably, the findings of this study are derived primarily from policy text analysis and therefore reflect the structure, orientation, and distribution of policy instruments within official policy documents, rather than the actual effectiveness of smart pharmaceutical regulation in practice. Existing implementation research has emphasized that policy design and policy text structure should not be directly equated with implementation effectiveness or policy outcomes, which require separate empirical assessment of implementation processes and outcomes (43). Consequently, the coding frequency of policy instruments in this study should be understood as an indicator of policy attention and institutional emphasis in policy design, rather than a direct measurement of regulatory performance or governance outcomes.

### 3.1 Basic selection and application of the policy instruments

Table 4 presents the distribution of policy instruments used in smart pharmaceutical regulation policies. Nodality-based instruments account for the largest share at 41.2%. Treasure-based instruments rank second with 24.1%, followed by authority-based instruments at 20.8%. Organization-based instruments constitute the smallest proportion at 13.9%. These results suggest that the policy texts of smart pharmaceutical regulation place comparatively greater emphasis on nodality-based instruments, whereas organization-based instruments receive relatively less textual emphasis.

**Table 4:** Distribution of policy instruments in smart pharmaceutical regulation.

Classification	Policy instruments (sub-nodes)	Number of source documents	Percentage (%)	Coded reference points	Percentage (%)
Nodality-based instruments	Information disclosure	55	61.1	146	5.7
	Risk surveillance	68	75.6	189	7.3
	Decision support	65	72.2	192	7.4
	Data collection and sharing	80	88.9	349	13.5
	Standardization	59	65.6	186	7.2
	Subtotal	88	97.8	1,062	41.2
Authority-based instruments	Licensing	49	54.4	85	3.3
	Traceability and compliance requirements	74	82.2	197	7.6
	Digital enforcement	63	70.0	123	4.8
	Risk control	43	47.8	75	2.9
	Sanctions	40	44.4	55	2.1
	Subtotal	86	95.6	535	20.8
Treasure-based instruments	Digital infrastructure	82	91.1	383	14.9
	Testing capacity	66	73.3	163	6.3
	Training funding	28	31.1	31	1.2
	Incentive subsidies	12	13.3	13	0.5
	Service outsourcing	23	25.5	32	1.2
	Subtotal	86	95.6	622	24.1
Organization-based instruments	Organizational structure	9	10.0	11	0.4
	Lifecycle regulation	53	58.9	134	5.2
	Interagency coordination	48	53.3	125	4.8
	Stakeholder co-governance	36	40.0	89	3.5
	Subtotal	68	75.6	359	13.9

“Coded reference points” indicates the frequency of coded segments associated with a category, reflecting the relative emphasis and thematic density of that instrument within policy texts. “Number of source documents” indicates the number of policy documents containing a given policy instrument category, reflecting the breadth of policy adoption across the corpus. The interpretation of policy instrument prominence in this study is based primarily on coded reference points, whereas the distribution of source documents serves as a complementary indicator of the breadth of policy adoption.

Nodality-based instruments are the most frequently used policy tools in smart pharmaceutical regulation policies. These instruments mainly focus on data collection and sharing, decision support, and risk surveillance. This pattern reflects that the ultimate objective of smart regulation in social regulatory domains is to prevent public safety incidents through quantifiable risk assessment, regulatory information sharing, effective risk communication, and data-driven regulatory approaches (44). Notably, data collection and sharing accounts for the largest proportion among all sub-nodes at 13.5%, highlighting the central role of data integration and information exchange in enabling data-driven pharmaceutical regulation.

Treasure-based instruments rank second among all policy instruments. In the context of smart pharmaceutical regulation, these instruments primarily support the development of digital regulatory infrastructure and the enhancement of technical capacities, reflecting strong policy attention to the financial and technological conditions associated with data-driven regulatory governance. Among the sub-nodes, digital infrastructure accounts for the largest share at 14.9%, reflecting that fiscal investment in smart platforms, databases, and regulatory information centers has become a central component within the policy text structure of smart pharmaceutical regulation. Testing capacity ranks second at 6.3%, indicating substantial policy attention to laboratory facilities and inspection centers. These findings suggest that treasure-based instruments in smart pharmaceutical regulation policies are primarily oriented toward infrastructure construction and technical-capacity enhancement. Conversely, the remaining sub-nodes account for much smaller proportions, suggesting that different types of financial and technical support receive varying degrees of policy emphasis.

Authority-based instruments rank third among all policy instruments, accounting for 20.8% of the total distribution. These instruments closely align with traditional command-and-control regulation, which has historically constituted the dominant regulatory approach in many social regulatory domains (45). Among the sub-nodes, traceability and compliance requirements account for the largest share at 7.6%, indicating that digital traceability and compliance-oriented regulation occupy a prominent position within the policy text structure of smart pharmaceutical regulation. In this context, Pharmaceutical Track-and-Trace Systems (PTTS) have emerged as essential tools for real-time monitoring, helping mitigate the risks of substandard and falsified medicines (SF medicines) and supporting end-to-end quality assurance across the pharmaceutical supply chain (46).

Organization-based instruments account for the smallest proportion among all policy instruments, representing 13.9% of the total distribution. These instruments mainly focus on institutional arrangements and organizational coordination to support the implementation of pharmaceutical regulation. Among the sub-nodes, lifecycle regulation accounts for the largest share at 5.2%, reflecting the growing emphasis on lifecycle-based pharmaceutical governance in China, where regulatory oversight increasingly extends across the entire lifecycle of medicines from production and circulation to post-market surveillance (47). This trend is reflected in recent legislative reforms. For example, the 2019 revision of the *Drug Administration Law* strengthened risk management, dynamic inspections, and post-market surveillance mechanisms, while the *Vaccine Management Law* established stricter regulatory controls covering the entire process from research and development to distribution. Interagency coordination follows with 4.8%, reflecting the increasing attention given to cross-departmental collaboration within the policy texts. Stakeholder co-governance accounts for 3.5%, suggesting an emerging but still limited role of social actors. Conversely, organizational structure appears only occasionally, suggesting that institutional restructuring has not been a primary focus in developing smart pharmaceutical regulation policies.

The observed distribution can be better understood when situated within the broader literature on NATO-based policy instrument analysis. Prior studies suggest that the relative prominence of each instrument type varies across policy domains, depending on the governance resources most central to the policy problem. For instance, in AI governance contexts, treasure-based instruments may be especially prominent, as governments often rely on public investment, financial incentives, and funding programs to stimulate technological development and capacity building (48). In digital government research, a study on digital government and circular economy transition found that authority- and treasure-based roles were more frequently identified than nodality- and organization-based roles, indicating that the configuration of government tools varies according to the policy problem and transition context (27). Similarly, studies of health-service organizations based on the NATO framework have found that authority- and organization-based instruments often occupy a dominant position, because governments tend to rely on administrative mandates, organizational integration, service-capacity building, and institutional coordination to promote healthcare reform and inter-organizational collaboration (29). Against this comparative background, the predominance of nodality-based instruments in this study reflects the data-intensive and platform-based nature of smart pharmaceutical regulation. At the same time, the comparatively low share of organization-based instruments suggests that institutional coordination and stakeholder co-governance receive less textual emphasis than data infrastructure and information-based regulatory tools.

### 3.2 Distribution of policy instruments across policy development stages

**Figure 3:**
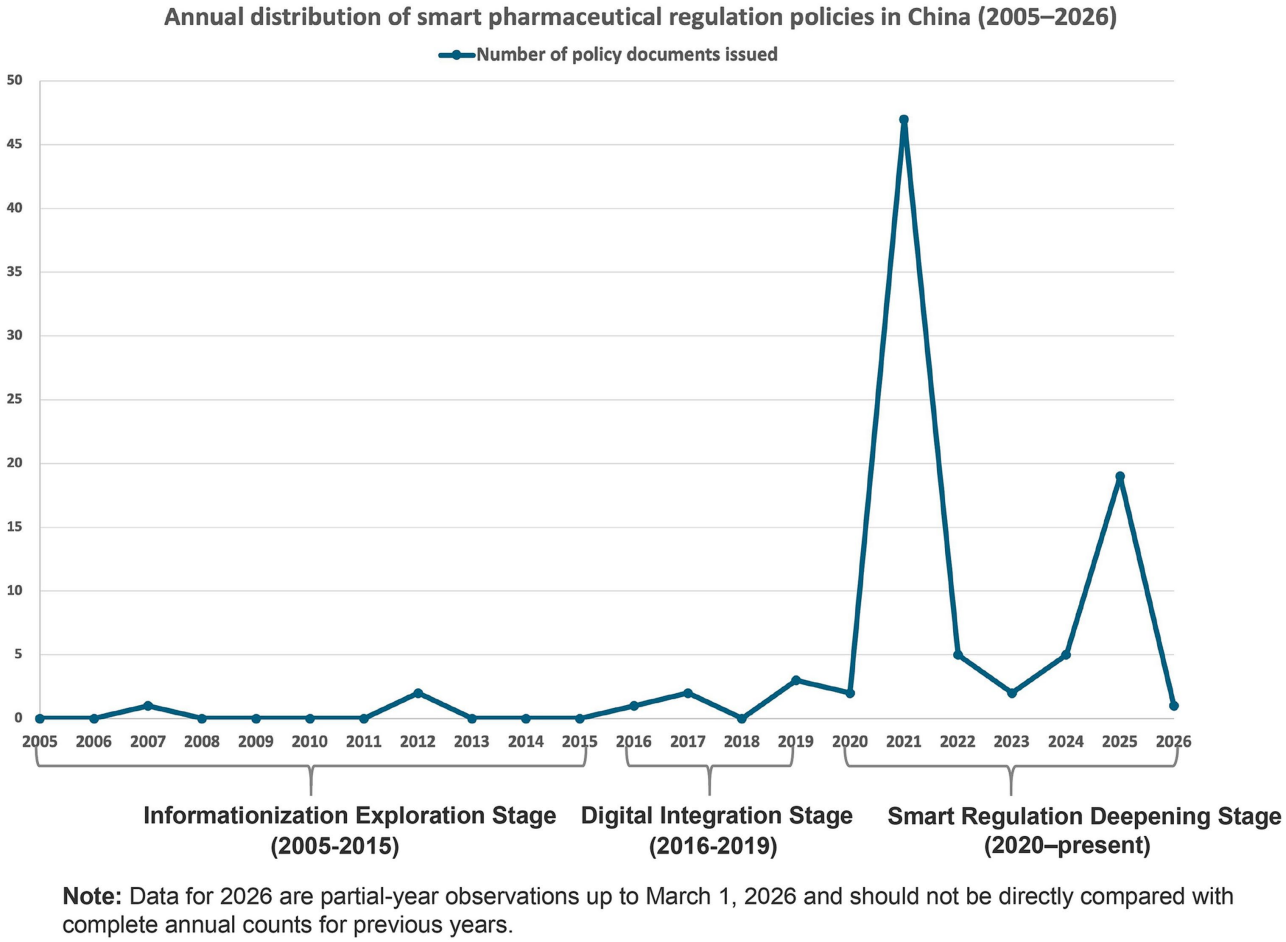
Annual distribution of smart pharmaceutical regulation policies in China (2005–2026).

In terms of the number of policy releases, among the 90 policy documents included in this study, 3 were issued during the informationization exploration stage (2005–2015), 6 during the digital integration stage (2016–2019), and 81 during the smart regulation deepening stage (2020–2026). This distribution indicates a significant increase in the number of policies related to smart pharmaceutical regulation over time. However, because the number of policy documents differs substantially across the three stages, the cross-stage comparison in this study is interpreted descriptively rather than as formal statistical inference. The stage-based analysis is used to contextualize the evolution of policy instruments and policy themes, rather than to infer causal effects from the volume of documents in each period. Figure 3 illustrates the number of policy documents across different years and stages. To further examine the use and characteristics of policy instruments across stages, the 90 policy documents were coded using NVivo matrix coding. In the matrix, the rows represent the NATO-based classification of policy instruments, while the columns correspond to the three policy development stages. Table 5 presents the distribution of policy instruments across stages obtained through this approach.

**Table 5:** Distribution of policy instruments across policy development stages.

Policy instruments	Informationization exploration stage (2005–2015)	Digital integration stage (2016–2019)	Smart regulation deepening stage (2020–present)
Nodality	18/38.3%	26/44.1%	1018/41.2%
Authority	8/17.0%	11/18.6%	516/20.8%
Treasure	14/29.8%	16/27.1%	592/24.0%
Organization	7/14.9%	6/10.2%	346/14.0%
Total	47/100%	59/100%	2472/100%

As shown in Table 5, the distribution of policy instruments varies across the three stages of smart pharmaceutical regulation. During the informationization exploration stage (2005–2015), nodality-based instruments accounted for the largest proportion at 38.3%, followed by treasure-based instruments at 29.8%. Authority-based and organization-based instruments were used less frequently. This pattern indicates that policy documents in the early stages of smart pharmaceutical regulation primarily focused on building regulatory information systems and digital infrastructure.

Smart pharmaceutical regulation can be understood as a sectoral application of e-government initiatives in this field. The emphasis on information infrastructure development during this stage aligns with the broader trajectory of e-government development, which typically begins with the establishment of online information systems and basic digital infrastructure before moving toward deeper integration and digital transformation (49). This trend is also reflected in early policy documents. For example, the *Drug Electronic Regulation Work Plan (2011–2015)* proposed constructing core infrastructure for pharmaceutical electronic supervision, including the pharmaceutical electronic supervision information platform and information security protection systems. The plan also emphasized the establishment of regulatory standards, such as business operation standards, data standards, and information security standards, as well as the development of a pharmaceutical supervision data center, data backup center, and electronic supervision service system (50). Despite the emphasis on information systems and digital infrastructure, smart pharmaceutical regulation had not yet established an integrated governance framework, but it laid the foundation for later integration.

During the digital integration stage (2016–2019), the share of nodality-based instruments continued to increase, remaining dominant at 44.1%. Authority-based instruments also gained importance, accounting for 18.6%, particularly regarding traceability requirements and mandatory compliance obligations. Meanwhile, treasure-based instruments continued to support the expansion of digital infrastructure and technical capacities. The distribution of policy instruments shifted toward a stronger emphasis on data integration and platform-based governance, indicating that smart pharmaceutical regulation entered a phase of integration, characterized by the development of regulatory platforms, data-sharing mechanisms, and traceability systems.

Before this stage, although smart regulatory platforms had been developed across different regions, problems such as data silos, lack of interoperability, and redundancies were prominent (51). Compared with the previous stage, regulatory systems became increasingly interconnected, enabling the exchange of information across different regulatory domains. Policy initiatives such as the promotion of “Internet Plus Regulation” and the establishment of pharmaceutical traceability systems further reinforced this trend. However, integration remained largely limited to data connectivity and platform construction. The coordination between regulatory actors and the effective use of integrated data for decision-making were still limited. As a result, smart pharmaceutical regulation had not yet fully transitioned into a mature, intelligence-driven governance model, but marked a critical step toward systematic integration and data-driven regulation.

During the smart regulation deepening stage (2020–present), nodality-based instruments remained dominant, accounting for 41.2%. The substantial increase in the proportion of the sub-node “decision support” within nodality-based instruments indicates that data were no longer used merely for information provision and sharing, but increasingly played a role in supporting regulatory decision-making. For example, the *Action Plan for Accelerating the Development of Smart Pharmaceutical Regulation* issued by the NMPA proposes strengthening data collection and sharing across the pharmaceutical supply chain through the national regulatory platform, integrating lifecycle data of pharmaceutical products, and establishing a decision-support system covering the entire regulatory process to support regulatory decision-making (16). Authority-based instruments further increased to 20.8%, reflecting the growing importance of digital enforcement and a transition toward enforcement practices increasingly driven by data and digital decision-making. For instance, the *List of Typical AI Application Scenarios in Pharmaceutical Regulation* issued by the NMPA demonstrates how artificial intelligence can support digital enforcement by automating compliance review, analyzing regulatory data, identifying non-compliance issues, and generating preliminary regulatory decisions (52). Organization-based instruments increased to 14.0%, with lifecycle regulation and interagency coordination taking on a central role, indicating that regulatory governance is moving toward a more integrated system that spans the entire lifecycle of pharmaceuticals and involves multiple regulatory actors.

Taken together, this stage marks the transition from data integration to increasingly intelligence-driven governance, characterized by the deep integration of data, decision-making, enforcement, and coordinated regulatory processes, indicating the emergence of a more mature and integrated stage of smart pharmaceutical regulation.

## 4 Discussion

### 4.1 Structural limitations of current smart pharmaceutical regulation

A key structural limitation of current smart pharmaceutical regulation, as reflected in the policy texts, is the underutilization of organization-based instruments, reflecting weak coordination both within government and between government and external stakeholders. From an aggregate perspective, organization-based instruments account for the lowest proportion among all policy instruments, at only 13.9%. From a temporal perspective, their share declined from 14.9% in the initial stage to 10.2% in the intermediate stage, then slightly increased to 14.0% in the most recent stage, yet still failing to exceed the initial level, indicating that they have not been consistently prioritized. At the sub-category level, both “organizational structure” and “stakeholder co-governance” remain relatively underrepresented, while “interagency coordination” is not the dominant sub-node. Taken together, these patterns point to persistent structural weaknesses in the deployment of organizational tools and reveal three interrelated dimensions of constraint, namely horizontal coordination within government, vertical coordination across levels, and co-governance with external stakeholders.

#### 4.1.1 Horizontal coordination challenges in smart pharmaceutical regulation

**Figure 4:**
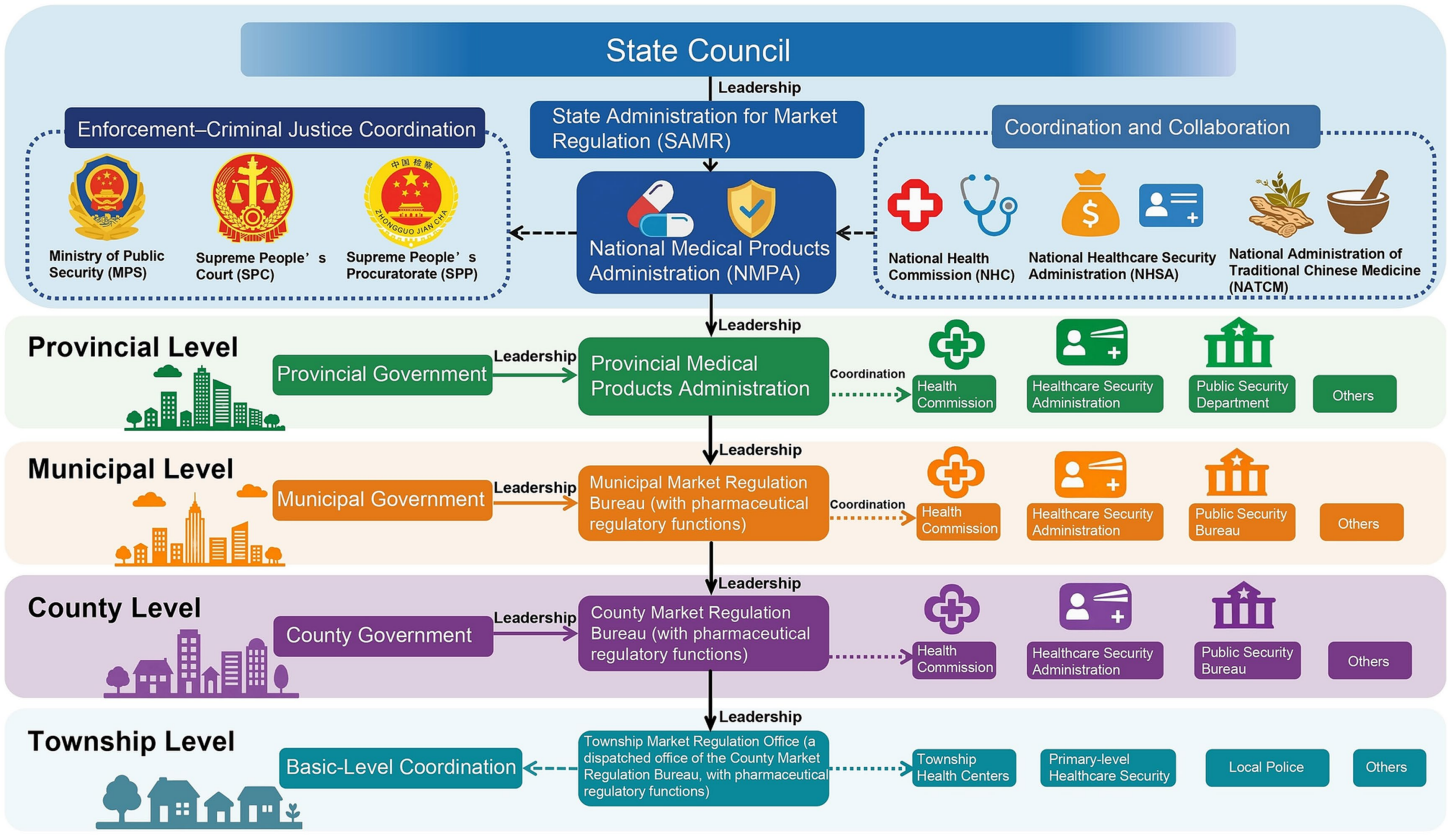
Multi-level governance structure of pharmaceutical regulation in China.

Horizontal coordination challenges in smart pharmaceutical regulation are primarily manifested in functional fragmentation and insufficient coordination mechanisms. Pharmaceutical regulation is a typical cross-sectoral and multi-level regulatory domain. Horizontally, it involves multiple governmental departments, while vertically it spans five administrative levels, including the central, provincial, municipal, county, and township levels, resulting in a highly complex organizational structure (see Figure 4). While smart regulation enhances traditional regulatory models, it also introduces new requirements for governance mechanisms, including data-driven governance through data standardization and information sharing (53), algorithmic governance through the application of analytical models and automated decision-making (54, 55), and platform-based governance supported by government cloud platforms and big data infrastructures (56). Evidence from the distribution of policy instruments indicates that substantial progress has been made in data integration and technological application. However, this technological advancement has not been matched by a corresponding organizational adaptation. In contexts characterized by high complexity, uncertainty, and interdependence, organizational structures are expected to evolve to support coordination and information processing (57). Yet, a contrasting pattern emerges at the organizational level.

Weber’s bureaucratic model enhances administrative specialization but also produces functional segmentation across departments, thereby increasing the difficulty of interagency coordination in smart pharmaceutical regulation (58). Pharmaceutical regulation covers the entire lifecycle, including research and development, production, distribution, and use, and lifecycle-based regulation is a central approach to smart pharmaceutical regulation (47). Yet, the current system remains functionally segmented, with different agencies responsible for distinct stages. Specifically, the NMPA oversees drug approval and registration; production and distribution are regulated through Good Manufacturing Practice (GMP) and Good Supply Practic*e* (GSP), enforced by regulatory authorities at different levels; clinical use is supervised by the National Health Commission (NHC); pricing and reimbursement are managed by the National Healthcare Security Administration (NHSA); and criminal enforcement involves coordination with public security authorities, procuratorates, and courts.

In terms of coordination mechanisms, China has established deliberative coordination bodies such as the Food and Drug Safety Committee, complemented by nationwide special enforcement campaigns. However, these mechanisms remain limited in both institutionalization and diversity. On the one hand, deliberative coordination bodies remain relatively limited in scope and institutional diversity. Existing food and drug safety coordination bodies are concentrated primarily at the provincial level, whereas a dedicated high-level coordination mechanism for pharmaceutical safety regulation is absent at the central level, making it difficult to support routine and complex cross-departmental coordination. On the other hand, cross-departmental coordination largely relies on special enforcement campaigns, which can be understood as a form of campaign-style governance characterized by the mobilization of administrative and social resources under political leadership and the establishment of multi-level command headquarters (59). However, such approaches tend to prioritize strict administrative directives over case-specific discretion, often resulting in disproportionately severe penalties and undermining the fairness of administrative discretion (44).

More importantly, these coordination arrangements largely extend traditional regulatory practices and have not evolved into new forms of horizontal coordination suited to smart regulation. This gap is reflected in structural deficiencies across three dimensions: regulatory agencies, powers, and obligations. First, there is no authoritative lead agency responsible for coordinating smart pharmaceutical regulation. In the absence of a clearly defined coordinating agency, different agencies act within their respective mandates and interests, leading to overlapping responsibilities, increased coordination costs, and potential interdepartmental conflicts, thereby weakening overall regulatory effectiveness.

Second, smart regulation requires adjustments to existing authority structures, yet institutional arrangements have not kept pace. As data sharing, algorithmic decision-making, and platform governance expand, the boundaries of authority in data access, analysis, and use have become increasingly complex. However, current regulations do not clearly define the scope of authority for different agencies in data governance and digital enforcement, resulting in ambiguity and uncertainty in practice.

Third, cross-departmental coordination responsibilities remain insufficiently institutionalized. Although policies repeatedly emphasize data sharing and collaborative governance, in practice, responsibilities for data reporting, information updating, and joint risk response remain unclear. The absence of binding constraints and effective incentives often renders coordination obligations largely symbolic.

#### 4.1.2 Vertical coordination challenges in smart pharmaceutical regulation

Vertical coordination in smart pharmaceutical regulation is constrained by disparities in resources and operational logic between central and local governments. First, differences in resource allocation between central and local governments constitute a key constraint on vertical coordination. As shown in Tables 6 and 7, provincial governments employ fewer nodality-based and organization-based instruments than the central level, with a particularly low proportion of the sub-node “organizational structure.” This suggests insufficient policy support for institutional restructuring at the provincial level, resulting in limited organizational capacity and institutional flexibility in smart regulation.

**Table 6:** Distribution of policy instruments in smart pharmaceutical regulation at the national and provincial levels.

Policy instruments	National level	Provincial level
Nodality	236/47.6%	826/39.7%
Authority	73/14.7%	462/22.2%
Treasure	112/22.6%	510/24.5%
Organization	75/15.1%	284/13.6%
Total	496/100%	2082/100%

**Table 7:** Distribution of sub-nodes of organization-based instruments at the national and provincial levels.

Sub-nodes of organization-based instruments	National level	Provincial level
Organizational structure	7/9.3%	4/1.4%
Lifecycle regulation	26/34.7%	108/38.1%
Interagency coordination	23/30.7%	102/35.9%
Stakeholder co-governance	19/25.3%	70/24.6%
Total	75/100%	284/100%

However, this gap does not simply indicate weaker digital capabilities at the local level. Rather, it reflects an asymmetry in resource distribution and platform control. Within China’s digital government system, key data infrastructures and information platforms are predominantly developed and operated at the central level. For example, the national government service platform and the national data sharing platform serve as central hubs for data aggregation, exchange, and access. Consequently, local governments rely heavily on centrally defined interfaces and standards when implementing data sharing and smart regulation, lacking the capacity to independently construct and integrate data resources.

Moreover, critical technological resources, such as artificial intelligence, cloud computing, and large-scale data centers, are concentrated at the central level and in a limited number of developed regions. This further reinforces the central government’s role as an information hub within the regulatory system. Under this configuration, the central government promotes nationwide data integration and platform development through nodality-based instruments, while provincial governments are largely confined to implementation and coordination functions, with limited scope for institutional innovation through organization-based instruments. As a result, vertical coordination is achieved in form through platform connectivity but lacks substantive institutional alignment based on balanced resources and compatible governance structures. The relative dependence of local governments on central resources constrains their ability to play a leading role in cross-level coordination, thereby exacerbating vertical misalignment in smart pharmaceutical regulation.

Second, central and local governments differ markedly in their operational logic in smart pharmaceutical regulation. This divergence can be understood through a layered governance perspective in digital government, in which functions, resources, and policy instruments are distributed asymmetrically across levels. The central government primarily performs a strategic role, while provincial governments combine strategic and hub functions, linking policy transmission with regional coordination (60).

In practice, the central level emphasizes institutional design, standard-setting, and platform development, seeking to build an integrated and system-wide regulatory framework through data integration and system architecture. Conversely, provincial governments, while implementing central strategies, also face concrete enforcement tasks and performance pressures. Their operational logic is therefore more execution-oriented, focusing on enforcement efficiency, risk response, and target fulfillment.

However, this system–execution division has not translated into a stable vertical coordination mechanism. On the one hand, the central logic of platform-based integration and data coordination is not proportionally institutionalized at the local level. On the other hand, the coordination capacity and institutional tools required for the provincial hub role remain underdeveloped, leading provincial governments to function more as channels for policy transmission and task decomposition than as coordinators.

For instance, the *Action Plan for Accelerating the Development of Smart Pharmaceutical Regulation* encourages local authorities to pilot traceability initiatives (16). In response, the *Hebei Provincial Medical Products Administration* issued a follow-up notice to increase enterprise participation and data quality in the pharmaceutical traceability system (61). This policy translated national objectives into measurable targets, such as coverage rates, data submission rates, and inspection completion rates, enforced through annual evaluations and special supervision. In this process, provincial governments primarily transmit and operationalize central policies by breaking them down into quantifiable tasks for lower levels. However, they play a limited role in cross-regional data integration and cross-departmental coordination, where institutional innovation and organization-based instruments remain insufficient.

As a result, a tension emerges between a system-oriented logic at the central level and an execution-oriented logic at the local level. While the former promotes integrated, data- and platform-based governance, the latter tends toward short-term, task-driven enforcement under bureaucratic and performance pressures. This divergence hinders the formation of a coherent governance model across levels and increases the complexity of vertical coordination.

#### 4.1.3 Co-governance challenges in smart pharmaceutical regulation

Beyond the horizontal and vertical coordination challenges within government, stakeholder co-governance accounts for only 3.5% of all coded reference points, indicating significant limitations in the development of social co-governance in smart pharmaceutical regulation.

Social co-governance refers to the collaborative formulation of laws and governance rules by enterprises, consumers, voters, non-governmental organizations, and other stakeholders (62). In China, it can be understood as a process in which multiple actors collectively manage public affairs based on social power, engaging in coordinated action to achieve shared interests through mechanisms such as consultative democracy (63). In the context of smart pharmaceutical regulation in China, stakeholders include, but are not limited to, government agencies, pharmaceutical manufacturers, wholesale and retail distributors, research institutions, industry associations, and consumers.

A central weakness lies in the limited participation and decision-making power of consumers. Public participation is a core element of democratic governance and a vital source of legitimacy for collaborative and regulatory governance (64). As the ultimate bearers of pharmaceutical safety risks, consumers should play a substantive role in governance. In practice, however, their participation is largely confined to passively receiving information disclosed by regulatory authorities, such as safety notices, risk alerts, and public education materials, supplemented by limited channels for complaints and reporting. Conversely, other stakeholders are more deeply embedded in the regulatory system. Universities and research institutions participate in establishing national or provincial key laboratories for drug testing, thereby contributing directly to regulatory capacity. Enterprises, meanwhile, influence regulatory rulemaking through their involvement in developing GMP standards and related technical norms. In comparison, consumers remain primarily information recipients and risk reporters. Although digital platforms enable them to provide feedback, their data contributions rarely translate into decision-making authority. This reflects an imbalance in the allocation of rights and responsibilities across stakeholders. While enterprises and research institutions are institutionally integrated into the regulatory system, consumers lack comparable channels for meaningful participation. If some stakeholders lack the capacity, organization, status, or resources to participate on an equal footing, governance processes are likely to be dominated or manipulated by more powerful actors (65).

### 4.2 International experience

The preceding analysis has identified major coordination deficits in China’s smart pharmaceutical regulation, including insufficient horizontal coordination among government departments, vertical coordination across administrative levels, and external coordination with stakeholders. Against this background, this section examines selected international experiences in pharmaceutical regulation and technology-enabled drug safety governance. The purpose is to better contextualize the coordination challenges in China and to identify potential institutional reference points for improving smart pharmaceutical regulation. The comparison focuses on the United States and the European Union (EU) because they represent two different but influential models of coordination in pharmaceutical safety regulation: a federal agency-led model based on formal agreements and a network-based model built around supranational coordination and national competent authorities. Table 8 summarizes the main differences among the United States, EU, and China’s approaches to coordination in smart pharmaceutical safety regulation.

**Table 8:** International comparison of coordination mechanisms in smart pharmaceutical regulation.

Dimension	United States framework	EU framework	China (current status)
Horizontal coordination	Formal inter-agency MOUs with CDC/NIH and intra-agency Intercenter Agreements among FDA product centers.	EMRN and HMA support coordination among EMA, national competent authorities, and the European Commission through information exchange, work-sharing, and regulatory consistency.	Coordination relies more on policy guidance, deliberative bodies, and campaign-style enforcement than routine issue-specific mechanisms.
Vertical coordination	Standard MOUs between the FDA and state pharmacy boards allocate responsibilities for compounded human drug products and interstate drug safety risks.	The EU pharmacovigilance system divides responsibilities between the EMA and national competent authorities; the EMA coordinates and harmonizes, while national authorities inspect and implement.	Central platforms and standards dominate; mechanisms for cross-level responsibility allocation, local feedback, and coordinated risk response remain insufficient.
External stakeholder co-governance	EDSTP/EDSTMs provide structured dialog with industry, academia, and technology stakeholders on AI-enabled pharmacovigilance.	EudraVigilance and the HMA-EMA AI workplan support data sharing, signal detection, and stakeholder engagement in AI-enabled medicines regulation.	Stakeholder participation is emerging but weakly institutionalized; consumers and external actors have limited channels for meaningful participation.

#### 4.2.1 Horizontal coordination

In the United States, horizontal coordination in pharmaceutical regulation is supported by both inter-agency and intra-agency mechanisms. At the inter-agency level, the FDA establishes formal cooperative arrangements with other federal public health agencies through Memoranda of Understanding (MOUs). For example, the FDA and the Centers for Disease Control and Prevention (CDC) have entered into an MOU that provides a framework for coordination and collaborative efforts, including information and data sharing, joint responses to public health issues, and coordinated communication before the issuance of major public advisories or publications (66). Similarly, FDA’s Center for Drug Evaluation and Research (CDER) maintains an MOU with the National Institutes of Health (NIH), which supports coordination and information sharing in drug safety oversight and helps connect regulatory decision-making with biomedical research expertise (67).

At the intra-agency level, the FDA also relies on Inter-center Agreements to coordinate regulatory responsibilities among its major product centers, such as CDER, the Center for Biologics Evaluation and Research (CBER), and the Center for Devices and Radiological Health (CDRH). These agreements are particularly important for products or regulatory issues that cross jurisdictional boundaries between drugs, biologics, and medical devices (68). Taken together, these mechanisms suggest that horizontal coordination in the United States pharmaceutical regulatory system is not limited to *ad hoc* administrative consultation. Instead, it is supported by formalized arrangements that define channels for information exchange, technical cooperation, and jurisdictional coordination across institutional boundaries.

In the EU, horizontal coordination is embedded in the European medicines regulatory network (EMRN), a closely coordinated network composed of national competent authorities in the European Economic Area (EEA), the European Medicines Agency (EMA), and the European Commission. EMA operates at the center of this network by coordinating interactions among more than 50 national competent authorities, whereas national authorities contribute experts to EMA’s scientific committees, working parties, and assessment teams. The Heads of Medicines Agencies (HMA) further supports horizontal coordination by promoting information exchange, IT development, best-practice sharing, regulatory consistency, and work-sharing across the network (69).

#### 4.2.2 Vertical coordination

Similar to horizontal coordination, vertical coordination in the United States is also supported by formalized MOUs between the FDA and state pharmacy boards or other appropriate state agencies concerning compounded human drug products. Under Section 503A of the *Federal Food, Drug, and Cosmetic Act* (FDCA), the Food and Drug Administration (FDA), in consultation with the National Association of Boards of Pharmacy (NABP), developed this standard MOU to manage the interstate distribution of compounded drugs and to guide state investigations of complaints regarding out-of-state distribution (70). The MOUs have been made available for state signature, with agreements concluded in states such as Kentucky and Colorado (71). These agreements allocate responsibilities between federal and state regulators: states investigate and respond to complaints, while the FDA maintains oversight of interstate drug safety risks. This mechanism illustrates a formalized model of vertical coordination that combines federal regulatory authority with state-level inspection, investigation, and enforcement capacity.

In the EU, vertical coordination is most evident in the pharmacovigilance system, which constitutes a central component of post-market drug safety governance and increasingly relies on data collection, signal detection, and coordinated risk response. EU law requires each marketing authorization holder, national competent authority, and EMA to operate a pharmacovigilance system, while the overall EU pharmacovigilance system functions through cooperation among EU Member States, EMA, and the European Commission; in some Member States, regional centers also operate under the coordination of national competent authorities (72). A more specific example is pharmacovigilance inspections, where national competent authorities are responsible for carrying out inspections, while EMA coordinates and harmonizes inspection activities at the Union level. This involves coordinating risk-based inspections, harmonizing guidance and technical interpretations, and supporting EU-wide regulatory consistency (73).

#### 4.2.3 Co-governance with external stakeholders

In the United States, co-governance with external stakeholders is supported by the FDA’s Emerging Drug Safety Technology Program (EDSTP), established within CDER. EDSTP serves as a central platform for dialog between CDER and industry, academic institutions, and technology providers on the use of AI and other emerging technologies in pharmacovigilance, while also supporting internal knowledge management and informing potential future regulatory and policy approaches (74).

This external coordination is operationalized through Emerging Drug Safety Technology Meetings (EDSTMs). These meetings provide eligible applicants and other relevant parties involved in pharmacovigilance, including those developing or applying AI-enabled tools, with an opportunity to discuss their research, development, and implementation of emerging technologies with CDER staff. Importantly, EDSTMs are intended to facilitate discussion and mutual learning rather than to promote a technology for FDA’s internal use or to provide formal regulatory advice on compliance with pharmacovigilance regulations (75). Taken together, the EDSTP and EDSTMs illustrate a relatively institutionalized model of co-governance with external stakeholders. Rather than treating industry, academic institutions, contract research organizations, pharmacovigilance vendors, and software developers merely as regulated objects, the FDA uses structured dialog to learn how emerging technologies are being applied in drug safety surveillance and to assess their risks, benefits, model evaluation processes, and implementation barriers.

Co-governance in the EU is supported by both pharmacovigilance data infrastructure and AI-related regulatory initiatives. EudraVigilance provides a shared database for suspected adverse drug reaction reports, and its access policy defines different levels of access for stakeholder groups. EMA and national competent authorities regularly review EudraVigilance data for signal detection, while the Pharmacovigilance Risk Assessment Committee (PRAC) evaluates detected safety signals and may recommend regulatory action (76). In the field of smart regulation, the HMA-EMA AI work plan to 2028 further adopts a collaborative and coordinated strategy for the use of AI in medicines regulation. It covers guidance, AI tools and technology, collaboration and training, experimentation, and states that regulators, medicine developers, academics, patient organizations, and other interested parties will be informed and engaged throughout implementation (77, 78).

#### 4.2.4 Comparative implications for China’s smart pharmaceutical regulation

The United States and EU experiences show that effective coordination in pharmaceutical safety regulation depends not only on digital platforms and data systems, but also on formalized institutional arrangements. The United States model represents a federal agency-led approach based on formal agreements, standard MOUs, and structured dialog with external stakeholders. The EU model represents a network-based approach built around the EMRN, the division of responsibilities between EMA and national competent authorities, shared pharmacovigilance infrastructures, and collaborative AI-related regulatory initiatives. Despite their institutional differences, both models demonstrate the importance of stable mechanisms for information exchange, responsibility allocation, technical harmonization, and stakeholder engagement.

Conversely, China’s smart pharmaceutical regulation has made significant progress in platform construction, data integration, traceability systems, and digital enforcement, but its coordination mechanisms remain comparatively underdeveloped. Horizontal coordination still relies heavily on general policy guidance and campaign-style enforcement; vertical coordination is shaped by central platforms and standards but lacks sufficient mechanisms for local feedback and for allocating cross-level responsibility; and stakeholder participation remains limited by the absence of stable co-governance channels. This comparison suggests that the next stage of China’s smart pharmaceutical regulation should focus on aligning technological tools with institutional design. Therefore, the following section proposes institutional pathways to strengthen horizontal coordination, vertical alignment, and stakeholder co-governance under a holistic government approach.

### 4.3 Institutional pathways for enhancing smart pharmaceutical regulation

The concept of holistic government provides a useful framework for addressing the coordination challenges identified in this study. It emphasizes the integration of fragmented governance through both horizontal and vertical coordination to improve policy effectiveness, reduce duplication, enhance resource efficiency, promote stakeholder collaboration, and deliver seamless public services (79). From this perspective, the application of organization-based instruments in smart pharmaceutical regulation should be reoriented toward a holistic governance approach. To operationalize this framework, future institutional reforms should focus on three interrelated dimensions: (1) diversifying horizontal coordination mechanisms; (2) reinforcing the service-oriented nature of smart pharmaceutical regulation; and (3) clarifying the allocation of authority and responsibilities among regulatory agencies and stakeholders.

#### 4.3.1 Diversifying horizontal coordination mechanisms

Policy fragmentation across regulatory domains should be reduced through more diversified horizontal coordination mechanisms. At present, inter-ministerial coordination within China’s central government has developed into three main forms: deliberative coordination bodies, inter-ministerial joint meetings, and departmental agreements (80). These existing arrangements can serve as a foundation for building a more comprehensive horizontal coordination framework for smart pharmaceutical regulation. For example, one possible institutional option is to establish a high-level deliberative coordination mechanism for smart pharmaceutical regulation, drawing on existing coordination arrangements within China’s central government. Such a mechanism might involve participation by key regulatory authorities, including the NMPA, the NHC, the State Administration for Market Regulation (SAMR), and the National Administration of Traditional Chinese Medicine (NATCM), thereby facilitating information sharing and reducing jurisdictional fragmentation across regulatory domains. Simultaneously, departmental agreements, characterized by flexibility and relatively low coordination costs, are particularly well suited for emerging and technically complex issues in smart pharmaceutical regulation, such as data governance and the allocation of data-related rights and responsibilities. The combined use of deliberative coordination bodies and departmental agreements can help establish a multi-layered toolbox of horizontal coordination instruments, thereby addressing the limitations in effectiveness and legality associated with existing mechanisms.

#### 4.3.2 Reinforcing the service-oriented nature of smart pharmaceutical regulation

The construction of public order and the provision of public services constitute two fundamental dimensions of modern administration. The New Public Service paradigm emphasizes that public administration should move from “steering” to “serving,” prioritizing public interest, citizen engagement, and service delivery (81). This shift reflects a broader reorientation of governmental functions from control-centered regulation toward service-oriented governance and provides an important theoretical foundation for the development of smart pharmaceutical regulation.

Although pharmaceutical regulation is traditionally viewed as a form of social regulation aimed at protecting public health (82), smart pharmaceutical regulation increasingly exhibits a dual character, combining regulatory control with public service functions in the context of digital and platform-based governance. For example, through real-time risk alerts and public information disclosure, platform-based compliance support for regulated entities, and lifecycle-oriented services that facilitate traceability and continuity of supervision. The dual character implies that smart pharmaceutical regulation should move beyond a narrow focus on enforcement and risk control toward the systematic integration of service-based functions. First, regulatory systems should strengthen information services by improving transparency, real-time risk communication, and public access to regulatory data. Second, platform-based services should be enhanced to support regulatory compliance, facilitate interagency coordination, and reduce transaction costs for regulated entities. Third, lifecycle-oriented regulatory services should be institutionalized to ensure continuity and coherence across different stages of pharmaceutical governance. Finally, greater attention should be given to user-oriented design to ensure that regulatory services are accessible, responsive, and aligned with the needs of diverse stakeholders.

#### 4.3.3 Allocating authority, rights, and responsibilities among regulatory actors

Effective coordination in smart pharmaceutical regulation requires a clear allocation of authority and responsibilities among regulatory actors. From an administrative law perspective, the configuration of rights and obligations serves as a core analytical lens and regulatory instrument, clarifying the legal status and functional roles of different actors and providing an institutional foundation for coordinated governance. Accordingly, this allocation should be structured along three interrelated dimensions.

First, the boundaries of authority among regulatory agencies should be clearly defined, particularly in areas such as data governance, algorithmic decision-making, and cross-departmental enforcement. As smart regulation increasingly relies on data integration and platform-based governance, unclear authority boundaries may lead to regulatory overlap, conflict, and coordination failure.

Second, the allocation of responsibilities should be institutionalized to ensure effective coordination. Regulatory actors at different levels should be assigned clearly defined duties in data reporting, information updating, risk identification, and joint response mechanisms. This requires moving beyond general policy statements toward enforceable institutional arrangements that specify accountability and reduce ambiguity in implementation.

Third, the framework should extend beyond government agencies to encompass multiple stakeholders, including enterprises, research institutions, and industry associations. As smart regulation is inherently multi-actor, effective governance depends on aligning the rights and obligations of different actors within a coherent institutional structure. This includes clarifying enterprises’ responsibilities regarding data disclosure and compliance, the role of technical institutions in supporting regulatory capacity, and the accountability of industry associations and third-party technical institutions for facilitating coordination.

More importantly, particular attention should be paid to addressing the imbalance in the allocation of rights and responsibilities among stakeholders, especially the marginal position of consumers. Mechanisms should be developed to enhance meaningful consumer participation, including expanding channels for public input, strengthening feedback integration into regulatory decision-making, ensuring that data contributed by users can effectively inform risk assessment and governance processes, and reinforcing consumers’ evaluative role in assessing the quality, accessibility, and responsiveness of smart pharmaceutical regulation as a public service.

### 4.4 Limitations and future research

#### 4.4.1 Limitations

This study has several limitations. First, it is based on policy text analysis and therefore primarily captures the institutional priorities and policy-instrument configurations reflected in official documents, rather than the actual implementation effects of smart pharmaceutical regulation. The frequency of coded policy instruments should thus be understood as an indicator of policy attention and design emphasis, not as a direct measure of regulatory performance.

Second, the policy corpus is limited to central- and provincial-level documents. Municipal-, county-, and township-level policies were excluded to maintain a relatively stable central–provincial analytical structure and to reduce duplication. However, lower-level policy documents may be important for understanding local implementation, institutional adaptation, and the practical operation of vertical coordination. Accordingly, the discussion of vertical coordination in this study should be interpreted mainly within the scope of central–provincial policy relations, rather than as a full account of all administrative levels.

Third, the distribution of policy documents across the three stages is highly uneven, with only three documents in the informationization exploration stage, six in the digital integration stage, and most documents concentrated in the smart regulation deepening stage. Although this pattern reflects the recent acceleration of smart pharmaceutical regulation policies in China, it limits the robustness of cross-stage comparison. Therefore, the stage-based findings should be interpreted descriptively rather than as statistically balanced comparisons. Moreover, coded reference counts may be affected by document length and coding granularity, and the 2026 data represent only partial-year observations up to March 1, 2026.

Finally, this study focuses on China as a single-country case. Its findings are shaped by China’s administrative structure, state-led digital governance model, and pharmaceutical regulatory system. Therefore, the conclusions should be generalized cautiously to other jurisdictions or regulatory contexts.

#### 4.4.2 Future research directions

First, future research could move beyond policy text analysis to examine how different policy instruments are used in actual regulatory practice and what regulatory effects they may produce. Second, the policy corpus could be expanded to include municipal-, county-, and township-level documents, thereby offering a more detailed understanding of local implementation, institutional adaptation, and grassroots regulatory practice. Third, future studies could apply a similar policy text content analysis approach to other jurisdictions, such as the United States, the European Union, or Japan, and systematically compare the configurations and evolution of smart pharmaceutical regulation instruments across different regulatory systems. Finally, further theoretical dialog with digital governance, algorithmic regulation, platform governance, and collaborative governance could help move the analysis beyond instrument classification toward a deeper understanding of how digital technologies reshape administrative discretion, institutional coordination, and regulatory legitimacy.

## 5 Conclusion

This study analyzed the evolution of smart pharmaceutical regulation in China using a two-dimensional framework combining policy instruments and policy development stages. The findings showed that nodality-based instruments, particularly those related to data collection, sharing, and decision support, dominate the policy landscape, reflecting strong policy emphasis on data-driven regulatory capacities. Treasure-based instruments provide important support through investments in digital infrastructure and technical capacity, whereas authority-based instruments continue to operate alongside emerging digital tools. Conversely, organization-based instruments receive comparatively less emphasis in policy texts.

From a temporal perspective, the policy texts reflect a shift from informationization to digital integration and toward intelligent regulation. Early policies focused on building basic information systems, followed by efforts to integrate data and establish platform-based supervision. At this stage, policy text increasingly emphasizes lifecycle-based governance and risk-oriented regulation supported by advanced technologies such as big data and artificial intelligence.

Based on these findings, this study suggests that under a holistic government approach, interagency coordination in smart pharmaceutical regulation could be enhanced by expanding the toolbox of horizontal coordination mechanisms. At the same time, clarifying the service-oriented nature of smart pharmaceutical regulation and ensuring a more balanced allocation of authority, rights, and responsibilities among multiple actors would contribute to more effective collaborative governance. Future research could complement policy text analysis with empirical studies on implementation and comparative analyses across different regulatory systems.
