# Supplementary material for: Policy text analysis of China’s smart pharmaceutical regulation under a policy tool–policy stage framework
Source: Front Public Health. 2026 Jun 17;14:1848335. doi: 10.3389/fpubh.2026.1848335 (PMC13319039; doi:10.3389/fpubh.2026.1848335)
Supplement: Supplementary file 1 [file Table_1.DOCX]

**Supplementary Material**

**Supplementary Tables**

Table S1. Policy documents on smart pharmaceutical regulation in China included in the analysis.

| **Number** | **Year** | **Chinese Title** | **English Title** | **Issuing Authority** | **Level** |
| --- | --- | --- | --- | --- | --- |
| 1 | 2007 | 国家食品药品安全“十一五”规划 | 11th Five-Year Plan for National Food and Drug Safety | General Office of the State Council | Central |
| 2 | 2012 | 2011—2015年药品电子监管工作规划 | Work Plan for Electronic Drug Regulation, 2011-2015 | State Food and Drug Administration | Central |
| 3 | 2017 | “十三五”国家药品安全规划 | 13th Five-Year National Drug Safety Plan | State Council | Central |
| 4 | 2019 | 关于加快推进药品智慧监管的行动计划 | Action Plan on Accelerating Smart Pharmaceutical Regulation | National Medical Products Administration | Central |
| 5 | 2022 | 药品监管网络安全与信息化建设“十四五”规划 | 14th Five-Year Plan for Cybersecurity and Informatization Development of Pharmaceutical Regulation | National Medical Products Administration | Central |
| 6 | 2021 | “十四五”国家药品安全及促进高质量发展规划 | 14th Five-Year National Plan for Drug Safety and Promotion of High-Quality Development | National Medical Products Administration et al. | Central |
| 7 | 2024 | 血液制品生产智慧监管三年行动计划(2024—2026年) | Three-Year Action Plan for Smart Regulation of Blood Product Production (2024-2026) | General Office of the National Medical Products Administration | Central |
| 8 | 2021 | 关于全面加强药品监管能力建设的实施意见 | Implementation Opinions on Comprehensively Strengthening Pharmaceutical Regulatory Capacity Building | General Office of the State Council | Central |
| 9 | 2024 | 药品监管人工智能典型应用场景清单 | List of Typical Artificial Intelligence Application Scenarios in Pharmaceutical Regulation | General Office of the National Medical Products Administration | Central |
| 10 | 2024 | 关于全面深化药品医疗器械监管改革促进医药产业高质量发展的意见 | Opinions on Comprehensively Deepening the Reform of Drug and Medical Device Regulation and Promoting High-Quality Development of the Pharmaceutical Industry | General Office of the State Council | Central |
| 11 | 2021 | “十四五”市场监管现代化规划的通知 | Notice on Issuing the 14th Five-Year Plan for Market Regulation Modernization | State Council | Central |
| 12 | 2019 | 关于建立职业化专业化药品检查员队伍的意见 | Opinions on Establishing a Professionalized and Specialized Drug Inspector Workforce | General Office of the State Council | Central |
| 13 | 2012 | 国家药品安全“十二五”规划 | 12th Five-Year Plan for National Drug Safety | State Council | Central |
| 14 | 2017 | 江西省人民政府关于贯彻落实“十三五”市场监管规划的实施意见 | Implementation Opinions of the Jiangxi Provincial People's Government on Implementing the 13th Five-Year Plan for Market Regulation | Jiangxi Provincial People's Government | Provincial |
| 15 | 2023 | 河北省人民政府关于加强数字政府建设的实施意见 | Implementation Opinions of the Hebei Provincial People's Government on Strengthening Digital Government Development | Hebei Provincial People's Government | Provincial |
| 16 | 2020 | 河北省药品监督管理局关于进一步提高药品流通领域药品追溯系统企业入网率和上传数据质量的通知 | Notice of the Hebei Medical Products Administration on Further Increasing Enterprise Enrollment in the Pharmaceutical Traceability System and Improving Uploaded Data Quality in Drug Distribution | Hebei Medical Products Administration | Provincial |
| 17 | 2016 | 山东省人民政府关于促进大数据发展的意见 | Opinions of the Shandong Provincial People's Government on Promoting Big Data Development | Shandong Provincial People's Government | Provincial |
| 18 | 2023 | 中共辽宁省委、辽宁省人民政府关于在辽宁全面振兴新突破三年行动中进一步保障和改善民生的实施意见 | Implementation Opinions of the CPC Liaoning Provincial Committee and Liaoning Provincial People's Government on Further Safeguarding and Improving People's Livelihoods in the Three-Year Action for New Breakthroughs in Liaoning's Comprehensive Revitalization | CPC Liaoning Provincial Committee; Liaoning Provincial People's Government | Provincial |
| 19 | 2021 | 湖南省人民政府办公厅关于全面加强药品监管能力建设的若干意见 | Several Opinions of the General Office of the Hunan Provincial People's Government on Comprehensively Strengthening Pharmaceutical Regulatory Capacity Building | General Office of the Hunan Provincial People's Government | Provincial |
| 20 | 2021 | 江苏省政府办公厅印发关于全面加强药品监管能力建设若干措施的通知 | Notice of the General Office of the Jiangsu Provincial People's Government on Issuing Several Measures for Comprehensively Strengthening Pharmaceutical Regulatory Capacity Building | General Office of the Jiangsu Provincial People's Government | Provincial |
| 21 | 2021 | 广东省人民政府办公厅关于印发广东省全面加强药品监管能力建设若干措施的通知 | Notice of the General Office of the Guangdong Provincial People's Government on Issuing Several Measures of Guangdong Province for Comprehensively Strengthening Pharmaceutical Regulatory Capacity Building | General Office of the Guangdong Provincial People's Government | Provincial |
| 22 | 2021 | 黑龙江省人民政府办公厅关于印发黑龙江省全面加强药品监管能力建设若干措施的通知 | Notice of the General Office of the Heilongjiang Provincial People's Government on Issuing Several Measures of Heilongjiang Province for Comprehensively Strengthening Pharmaceutical Regulatory Capacity Building | General Office of the Heilongjiang Provincial People's Government | Provincial |
| 23 | 2021 | 湖北省人民政府办公厅关于印发湖北省全面加强药品监管能力建设实施方案的通知 | Notice of the General Office of the Hubei Provincial People's Government on Issuing the Implementation Plan of Hubei Province for Comprehensively Strengthening Pharmaceutical Regulatory Capacity Building | General Office of the Hubei Provincial People's Government | Provincial |
| 24 | 2022 | 上海市人民政府办公厅印发《关于全面加强药品监管能力建设的实施意见》的通知 | Notice of the General Office of the Shanghai Municipal People's Government on Issuing the Implementation Opinions on Comprehensively Strengthening Pharmaceutical Regulatory Capacity Building | General Office of the Shanghai Municipal People's Government | Provincial |
| 25 | 2021 | 重庆市人民政府办公厅关于印发重庆市全面加强药品监管能力建设若干措施的通知 | Notice of the General Office of the Chongqing Municipal People's Government on Issuing Several Measures of Chongqing Municipality for Comprehensively Strengthening Pharmaceutical Regulatory Capacity Building | General Office of the Chongqing Municipal People's Government | Provincial |
| 26 | 2021 | 天津市人民政府办公厅印发关于全面加强药品监管能力建设实施方案的通知 | Notice of the General Office of the Tianjin Municipal People's Government on Issuing the Implementation Plan for Comprehensively Strengthening Pharmaceutical Regulatory Capacity Building | General Office of the Tianjin Municipal People's Government | Provincial |
| 27 | 2021 | 山东省人民政府关于全面加强药品监管能力建设若干措施的通知 | Notice of the Shandong Provincial People's Government on Several Measures for Comprehensively Strengthening Pharmaceutical Regulatory Capacity Building | Shandong Provincial People's Government | Provincial |
| 28 | 2021 | 浙江省人民政府办公厅关于全面加强省域药品监管能力建设的实施意见 | Implementation Opinions of the General Office of the Zhejiang Provincial People's Government on Comprehensively Strengthening Provincial Pharmaceutical Regulatory Capacity Building | General Office of the Zhejiang Provincial People's Government | Provincial |
| 29 | 2021 | 河南省人民政府办公厅关于印发河南省全面加强药品监管能力建设若干措施的通知 | Notice of the General Office of the Henan Provincial People's Government on Issuing Several Measures of Henan Province for Comprehensively Strengthening Pharmaceutical Regulatory Capacity Building | General Office of the Henan Provincial People's Government | Provincial |
| 30 | 2021 | 福建省药品安全和产业促进领导小组印发关于加强药品监管和产业促进能力建设实施方案的通知 | Notice of the Fujian Provincial Leading Group for Drug Safety and Industry Promotion on Issuing the Implementation Plan for Strengthening Pharmaceutical Regulation and Industry Promotion Capacity Building | Fujian Provincial Leading Group for Drug Safety and Industry Promotion | Provincial |
| 31 | 2021 | 河北省人民政府办公厅印发关于全面加强药品监管能力建设若干措施的通知 | Notice of the General Office of the Hebei Provincial People's Government on Issuing Several Measures for Comprehensively Strengthening Pharmaceutical Regulatory Capacity Building | General Office of the Hebei Provincial People's Government | Provincial |
| 32 | 2019 | 中共吉林省委办公厅、吉林省政府办公厅印发《关于全面加强药品安全监管工作的意见》 | Notice of the General Office of the CPC Jilin Provincial Committee and the General Office of the Jilin Provincial People's Government on Issuing the Opinions on Comprehensively Strengthening Drug Safety Regulation | General Office of the CPC Jilin Provincial Committee; General Office of the Jilin Provincial People's Government | Provincial |
| 33 | 2021 | 吉林省人民政府办公厅关于印发全面加强药品监管能力建设若干措施的通知 | Notice of the General Office of the Jilin Provincial People's Government on Issuing Several Measures for Comprehensively Strengthening Pharmaceutical Regulatory Capacity Building | General Office of the Jilin Provincial People's Government | Provincial |
| 34 | 2022 | 内蒙古自治区人民政府办公厅关于全面加强药品监管能力建设工作措施的通知 | Notice of the General Office of the People's Government of Inner Mongolia Autonomous Region on Work Measures for Comprehensively Strengthening Pharmaceutical Regulatory Capacity Building | General Office of the People's Government of Inner Mongolia Autonomous Region | Provincial |
| 35 | 2021 | 宁夏回族自治区人民政府办公厅关于全面加强药品监管能力建设的实施意见 | Implementation Opinions of the General Office of the People's Government of Ningxia Hui Autonomous Region on Comprehensively Strengthening Pharmaceutical Regulatory Capacity Building | General Office of the People's Government of Ningxia Hui Autonomous Region | Provincial |
| 36 | 2021 | 山西省人民政府办公厅关于全面加强药品监管能力建设的通知 | Notice of the General Office of the Shanxi Provincial People's Government on Comprehensively Strengthening Pharmaceutical Regulatory Capacity Building | General Office of the Shanxi Provincial People's Government | Provincial |
| 37 | 2021 | 青海省人民政府关于进一步加强药品安全监管工作的意见 | Opinions of the Qinghai Provincial People's Government on Further Strengthening Drug Safety Regulation | Qinghai Provincial People's Government | Provincial |
| 38 | 2021 | 新疆维吾尔自治区人民政府印发关于全面加强药品监管能力建设的实施意见的通知 | Notice of the People's Government of Xinjiang Uygur Autonomous Region on Issuing the Implementation Opinions on Comprehensively Strengthening Pharmaceutical Regulatory Capacity Building | People's Government of Xinjiang Uygur Autonomous Region | Provincial |
| 39 | 2021 | 西藏自治区人民政府办公厅关于印发2021—2022年贯彻落实《国务院办公厅关于全面加强药品监管能力建设的实施意见》推进计划的通知 | Notice of the General Office of the People's Government of Tibet Autonomous Region on Issuing the 2021-2022 Promotion Plan for Implementing the Implementation Opinions of the General Office of the State Council on Comprehensively Strengthening Pharmaceutical Regulatory Capacity Building | General Office of the People's Government of Tibet Autonomous Region | Provincial |
| 40 | 2022 | 云南省人民政府办公厅关于印发云南省全面加强药品监管能力建设22条措施的通知 | Notice of the General Office of the Yunnan Provincial People's Government on Issuing 22 Measures of Yunnan Province for Comprehensively Strengthening Pharmaceutical Regulatory Capacity Building | General Office of the Yunnan Provincial People's Government | Provincial |
| 41 | 2021 | 广西壮族自治区人民政府办公厅关于印发广西全面加强药品监管能力建设实施方案的通知 | Notice of the General Office of the People's Government of Guangxi Zhuang Autonomous Region on Issuing the Implementation Plan of Guangxi for Comprehensively Strengthening Pharmaceutical Regulatory Capacity Building | General Office of the People's Government of Guangxi Zhuang Autonomous Region | Provincial |
| 42 | 2021 | 贵州省人民政府办公厅关于印发贵州省全面加强药品监管能力建设若干措施的通知 | Notice of the General Office of the Guizhou Provincial People's Government on Issuing Several Measures of Guizhou Province for Comprehensively Strengthening Pharmaceutical Regulatory Capacity Building | General Office of the Guizhou Provincial People's Government | Provincial |
| 43 | 2021 | 江西省人民政府办公厅关于印发全面加强药品监管能力建设若干措施的通知 | Notice of the General Office of the Jiangxi Provincial People's Government on Issuing Several Measures for Comprehensively Strengthening Pharmaceutical Regulatory Capacity Building | General Office of the Jiangxi Provincial People's Government | Provincial |
| 44 | 2021 | 甘肃省人民政府办公厅关于印发全面加强药品监管能力建设促进医药产业高质量发展若干措施的通知 | Notice of the General Office of the Gansu Provincial People's Government on Issuing Several Measures for Comprehensively Strengthening Pharmaceutical Regulatory Capacity Building and Promoting High-Quality Development of the Pharmaceutical Industry | General Office of the Gansu Provincial People's Government | Provincial |
| 45 | 2021 | 陕西省人民政府办公厅关于印发全面加强药品监管能力建设若干措施的通知 | Notice of the General Office of the Shaanxi Provincial People's Government on Issuing Several Measures for Comprehensively Strengthening Pharmaceutical Regulatory Capacity Building | General Office of the Shaanxi Provincial People's Government | Provincial |
| 46 | 2025 | 安徽省全面深化药品医疗器械监管改革 促进医药产业高质量发展若干措施 | Several Measures of Anhui Province for Comprehensively Deepening the Reform of Drug and Medical Device Regulation and Promoting High-Quality Development of the Pharmaceutical Industry | Anhui Medical Products Administration et al. | Provincial |
| 47 | 2025 | 江西省人民政府办公厅关于全面深化药品医疗器械化妆品监管改革促进医药产业高质量发展的实施意见 | Implementation Opinions of the General Office of the Jiangxi Provincial People's Government on Comprehensively Deepening the Reform of Drug, Medical Device, and Cosmetics Regulation and Promoting High-Quality Development of the Pharmaceutical Industry | General Office of the Jiangxi Provincial People's Government | Provincial |
| 48 | 2025 | 上海市全面深化药品医疗器械监管改革促进医药产业高质量发展的若干措施 | Several Measures of Shanghai Municipality for Comprehensively Deepening the Reform of Drug and Medical Device Regulation and Promoting High-Quality Development of the Pharmaceutical Industry | General Office of the Shanghai Municipal People's Government | Provincial |
| 49 | 2024 | 天津市药品监督管理局关于印发深入贯彻党的二十届三中全会精神进一步全面深化药品监管领域改革若干措施的通知 | Notice of the Tianjin Medical Products Administration on Issuing Several Measures for Thoroughly Implementing the Spirit of the Third Plenary Session of the 20th CPC Central Committee and Further Comprehensively Deepening Reform in Pharmaceutical Regulation | Tianjin Medical Products Administration | Provincial |
| 50 | 2026 | 重庆市深化药品医疗器械监管改革促进医药产业高质量发展若干措施 | Several Measures of Chongqing Municipality for Deepening the Reform of Drug and Medical Device Regulation and Promoting High-Quality Development of the Pharmaceutical Industry | General Office of the Chongqing Municipal People's Government | Provincial |
| 51 | 2025 | 海南省全面深化药品医疗器械化妆品监管改革促进医药产业高质量发展实施方案 | Implementation Plan of Hainan Province for Comprehensively Deepening the Reform of Drug, Medical Device, and Cosmetics Regulation and Promoting High-Quality Development of the Pharmaceutical Industry | General Office of the Hainan Provincial People's Government | Provincial |
| 52 | 2025 | 江苏省政府办公厅印发关于全面推进药品医疗器械监管深层次改革促进医药产业高质量发展若干政策措施的通知 | Notice of the General Office of the Jiangsu Provincial People's Government on Issuing Several Policy Measures for Comprehensively Advancing In-Depth Reform of Drug and Medical Device Regulation and Promoting High-Quality Development of the Pharmaceutical Industry | General Office of the Jiangsu Provincial People's Government | Provincial |
| 53 | 2025 | 山东省人民政府办公厅关于全面深化药品医疗器械监管改革促进医药产业高质量发展的实施意见 | Implementation Opinions of the General Office of the Shandong Provincial People's Government on Comprehensively Deepening the Reform of Drug and Medical Device Regulation and Promoting High-Quality Development of the Pharmaceutical Industry | General Office of the Shandong Provincial People's Government | Provincial |
| 54 | 2025 | 浙江省全面深化药品医疗器械监管改革促进医药产业高质量发展的实施意见 | Implementation Opinions of Zhejiang Province on Comprehensively Deepening the Reform of Drug and Medical Device Regulation and Promoting High-Quality Development of the Pharmaceutical Industry | Zhejiang Medical Products Administration et al. | Provincial |
| 55 | 2025 | 四川省支持生物医药和医疗器械产业高质量发展若干政策措施 | Several Policy Measures of Sichuan Province to Support High-Quality Development of the Biomedical and Medical Device Industries | General Office of the Sichuan Provincial People's Government | Provincial |
| 56 | 2024 | 湖北省药监局印发《进一步发挥监管职能促进医药产业高质量发展若干措施》（修订稿） | Hubei Medical Products Administration Issued the Several Measures for Further Leveraging Regulatory Functions to Promote High-Quality Development of the Pharmaceutical Industry (Revised Draft) | Hubei Medical Products Administration | Provincial |
| 57 | 2025 | 湖南省创新药品耗材全域智慧管理模式的实施方案 | Implementation Plan of Hunan Province for an Innovative Region-Wide Smart Management Model for Drugs and Medical Consumables | Hunan Healthcare Security Administration et al. | Provincial |
| 58 | 2025 | 福建省药品监督管理局等十一部门印发《贯彻落实＜关于全面深化药品医疗器械监管改革促进医药产业高质量发展的意见＞的工作方案》的通知 | Notice of the Fujian Medical Products Administration and Ten Other Departments on Issuing the Work Plan for Implementing the Opinions on Comprehensively Deepening the Reform of Drug and Medical Device Regulation and Promoting High-Quality Development of the Pharmaceutical Industry | Fujian Medical Products Administration et al. | Provincial |
| 59 | 2021 | 贵州省药品监督管理局印发《关于助推医药产业高质量发展的若干措施》的通知 | Notice of the Guizhou Medical Products Administration on Issuing Several Measures to Promote High-Quality Development of the Pharmaceutical Industry | Guizhou Medical Products Administration | Provincial |
| 60 | 2025 | 黑龙江省药品监督管理局关于发布全面深化药品医疗器械监管改革促进医药产业高质量发展若干措施的公告 | Announcement of the Heilongjiang Medical Products Administration on Issuing Several Measures for Comprehensively Deepening the Reform of Drug and Medical Device Regulation and Promoting High-Quality Development of the Pharmaceutical Industry | Heilongjiang Medical Products Administration | Provincial |
| 61 | 2025 | 辽宁省药品监督管理局、辽宁省工业和信息化厅关于加快推进辽宁省生物医药产业数智化转型升级的实施意见 | Implementation Opinions of the Liaoning Medical Products Administration and the Liaoning Department of Industry and Information Technology on Accelerating Digital and Intelligent Transformation and Upgrading of Liaoning's Biomedical Industry | Liaoning Medical Products Administration; Liaoning Department of Industry and Information Technology | Provincial |
| 62 | 2025 | 内蒙古自治区药品监督管理局关于印发深化化妆品监管改革促进产业高质量发展的若干措施的通知 | Notice of the Inner Mongolia Medical Products Administration on Issuing Several Measures for Deepening Cosmetics Regulatory Reform and Promoting High-Quality Industrial Development | Inner Mongolia Medical Products Administration | Provincial |
| 63 | 2025 | 内蒙古自治区药品监督管理局关于印发深化药品医疗器械监管改革促进医药产业高质量发展若干措施的通知 | Notice of the Inner Mongolia Medical Products Administration on Issuing Several Measures for Deepening the Reform of Drug and Medical Device Regulation and Promoting High-Quality Development of the Pharmaceutical Industry | Inner Mongolia Medical Products Administration | Provincial |
| 64 | 2025 | 宁夏回族自治区关于全面深化药品医疗器械监管改革促进医药产业高质量发展的若干措施 | Several Measures of Ningxia Hui Autonomous Region for Comprehensively Deepening the Reform of Drug and Medical Device Regulation and Promoting High-Quality Development of the Pharmaceutical Industry | General Office of the People's Government of Ningxia Hui Autonomous Region | Provincial |
| 65 | 2025 | 山西省药品监督管理局等12部门关于印发山西省全面深化药品医疗器械监管改革促进医药产业高质量发展若干措施(试行)的通知 | Notice of the Shanxi Medical Products Administration and 11 Other Departments on Issuing Several Measures of Shanxi Province for Comprehensively Deepening the Reform of Drug and Medical Device Regulation and Promoting High-Quality Development of the Pharmaceutical Industry (Trial) | Shanxi Medical Products Administration et al. | Provincial |
| 66 | 2025 | 云南省全面深化药品医疗器械监管改革促进医药产业高质量发展十九条措施 | Nineteen Measures of Yunnan Province for Comprehensively Deepening the Reform of Drug and Medical Device Regulation and Promoting High-Quality Development of the Pharmaceutical Industry | Yunnan Medical Products Administration et al. | Provincial |
| 67 | 2025 | 广西全面深化药品医疗器械监管改革促进医药产业高质量发展行动方案(2025―2027年) | Action Plan of Guangxi for Comprehensively Deepening the Reform of Drug and Medical Device Regulation and Promoting High-Quality Development of the Pharmaceutical Industry (2025-2027) | General Office of the People's Government of Guangxi Zhuang Autonomous Region | Provincial |
| 68 | 2021 | 安徽省“十四五”药品安全发展规划 | 14th Five-Year Plan of Anhui Province for Drug Safety Development | General Office of the Anhui Provincial People's Government | Provincial |
| 69 | 2021 | 甘肃省“十四五”药品安全和高质量发展规划 | 14th Five-Year Plan of Gansu Province for Drug Safety and High-Quality Development | Gansu Medical Products Administration | Provincial |
| 70 | 2021 | 江西省“十四五”药品安全及高质量发展规划 | 14th Five-Year Plan of Jiangxi Province for Drug Safety and High-Quality Development | Jiangxi Medical Products Administration | Provincial |
| 71 | 2021 | 上海市药品安全和高质量发展“十四五”规划 | 14th Five-Year Plan of Shanghai Municipality for Drug Safety and High-Quality Development | Shanghai Medical Products Administration | Provincial |
| 72 | 2021 | 贵州省“十四五”药品安全及高质量发展规划 | 14th Five-Year Plan of Guizhou Province for Drug Safety and High-Quality Development | Guizhou Medical Products Administration | Provincial |
| 73 | 2021 | 重庆市药品安全及高质量发展“十四五”规划（2021—2025年） | 14th Five-Year Plan of Chongqing Municipality for Drug Safety and High-Quality Development (2021-2025) | General Office of the Chongqing Municipal People's Government | Provincial |
| 74 | 2021 | 广东省药品安全及高质量发展“十四五”规划（2021-2025 年） | 14th Five-Year Plan of Guangdong Province for Drug Safety and High-Quality Development (2021-2025) | Guangdong Medical Products Administration; Guangdong Development and Reform Commission | Provincial |
| 75 | 2021 | 陕西省“十四五”药品安全及高质量发展规划的通知 | Notice on the 14th Five-Year Plan of Shaanxi Province for Drug Safety and High-Quality Development | Shaanxi Medical Products Administration; Shaanxi Development and Reform Commission | Provincial |
| 76 | 2021 | 山东省“十四五”药品安全规划 | 14th Five-Year Plan of Shandong Province for Drug Safety | General Office of the Shandong Provincial People's Government | Provincial |
| 77 | 2021 | 浙江省药品安全“十四五”规划 | 14th Five-Year Plan of Zhejiang Province for Drug Safety | Zhejiang Development and Reform Commission; Zhejiang Medical Products Administration | Provincial |
| 78 | 2021 | 四川省“十四五”药品安全及高质量发展规划 | 14th Five-Year Plan of Sichuan Province for Drug Safety and High-Quality Development | Sichuan Medical Products Administration | Provincial |
| 79 | 2022 | 河南省“十四五”药品安全规划 | 14th Five-Year Plan of Henan Province for Drug Safety | Henan Administration for Market Regulation; Henan Medical Products Administration | Provincial |
| 80 | 2021 | 湖北省药品安全及促进医药产业高质量发展“十四五”规划 | 14th Five-Year Plan of Hubei Province for Drug Safety and Promotion of High-Quality Development of the Pharmaceutical Industry | General Office of the Hubei Provincial People's Government | Provincial |
| 81 | 2021 | 湖南省“十四五”药品安全规划 | 14th Five-Year Plan of Hunan Province for Drug Safety | Hunan Medical Products Administration | Provincial |
| 82 | 2021 | 福建省“十四五”药品安全及服务产业高质量发展规划 | 14th Five-Year Plan of Fujian Province for Drug Safety and Serving High-Quality Industrial Development | Fujian Provincial Leading Group for Drug Safety and Industry Promotion | Provincial |
| 83 | 2021 | 河北省食品药品安全监管“十四五”规划 | 14th Five-Year Plan of Hebei Province for Food and Drug Safety Regulation | Hebei Administration for Market Regulation | Provincial |
| 84 | 2021 | 黑龙江省“十四五”药品安全及高质量发展规划 | 14th Five-Year Plan of Heilongjiang Province for Drug Safety and High-Quality Development | General Office of the Heilongjiang Provincial People's Government | Provincial |
| 85 | 2021 | 吉林省药品安全及促进高质量发展“十四五”规划的通知 | Notice on the 14th Five-Year Plan of Jilin Province for Drug Safety and Promotion of High-Quality Development | Jilin Medical Products Administration | Provincial |
| 86 | 2021 | 辽宁省“十四五”药品安全规划 | 14th Five-Year Plan of Liaoning Province for Drug Safety | Liaoning Administration for Market Regulation; Liaoning Medical Products Administration; Liaoning Intellectual Property Administration | Provincial |
| 87 | 2021 | 内蒙古自治区“十四五”药品安全及高质量发展规划 | 14th Five-Year Plan of Inner Mongolia Autonomous Region for Drug Safety and High-Quality Development | Inner Mongolia Administration for Market Regulation | Provincial |
| 88 | 2021 | 宁夏药品安全及高质量发展“十四五”规划 | 14th Five-Year Plan of Ningxia for Drug Safety and High-Quality Development | Ningxia Medical Products Administration | Provincial |
| 89 | 2021 | 西藏自治区药品安全及高质量发展“十四五”规划 | 14th Five-Year Plan of Tibet Autonomous Region for Drug Safety and High-Quality Development | Tibet Medical Products Administration | Provincial |
| 90 | 2021 | 云南省“十四五” 药品安全规划 | 14th Five-Year Plan of Yunnan Province for Drug Safety | Yunnan Administration for Market Regulation; Yunnan Medical Products Administration | Provincial |

Supplementary Table S2. Representative examples of coding rules and coded policy segments

| **Policy ID** | **Policy title** | **Coded policy segment** | **Location in document** | **First-level node** | **Sub-node** | **Coding rationale** |
| --- | --- | --- | --- | --- | --- | --- |
| 14 | Implementation Opinions of the Jiangxi Provincial People's Government on Implementing the 13th Five-Year Plan for Market Regulation | Promote the aggregation and integration of market regulation data, statutory inspection and testing data, data on violations and dishonesty, and complaint/reporting data in areas such as workplace safety, environmental governance, food and drug safety, financial security, and tax administration, and disclose them to the public in a timely manner. | p. 13, Chapter 3, Section 2, lines 4–6 | Nodality-based instruments | Information disclosure | This segment emphasizes the timely public disclosure of integrated regulatory information. Its dominant governance function is to disseminate information and reduce information asymmetry, which corresponds to nodality-based instruments and the sub-node of information disclosure. |
| 22 | Notice of the General Office of the Heilongjiang Provincial People's Government on Issuing Several Measures of Heilongjiang Province for Comprehensively Strengthening Pharmaceutical Regulatory Capacity Building | Implement disciplinary measures for serious acts of dishonesty in accordance with laws and regulations. | p. 5, Chapter 2, Section 14, lines 6–7 | Authority-based instruments | Sanctions | This segment requires legally based disciplinary measures against serious untrustworthy conduct. Its dominant governance function is the use of formal regulatory authority to impose punitive or corrective consequences on non-compliant actors, which corresponds to authority-based instruments and the sub-node of sanctions. |
| 25 | Notice of the General Office of the Chongqing Municipal People's Government on Issuing Several Measures of Chongqing Municipality for Comprehensively Strengthening Pharmaceutical Regulatory Capacity Building | Accelerate the introduction of technologies and the allocation of equipment, and explore the use of third-party professional technical resources to monitor online drug sales. | p. 4, Chapter 4, Section 14, lines 2–3 | Treasure-based instruments | Service outsourcing | This segment focuses on relying on third-party professional technical capacity to support monitoring of online drug sales. Its dominant governance function is to mobilize external technical services and resources for regulatory implementation, which corresponds to treasure-based instruments and the sub-node of service outsourcing. |
| 57 | Implementation Plan of Hunan Province for an Innovative Region-Wide Smart Management Model for Drugs and Medical Consumables | The provincial healthcare security, pharmaceutical regulatory, and health administrative departments shall strengthen joint supervision in accordance with laws and regulations, achieving the sharing of management rules, exchange of information leads, coordinated supervision and enforcement, and joint investigation, punishment, and disciplinary action. | p. 2, Chapter 2, Section 4, lines 1–2 | Organization-based instruments | Interagency coordination | This segment establishes joint supervision among healthcare security, pharmaceutical regulation, and health administration departments. Its dominant governance function is to coordinate responsibilities, information exchange, enforcement actions, and joint disciplinary measures across agencies, which corresponds to organization-based instruments and the sub-node of interagency coordination. |

**Note.** The coded policy segments are translated from the original Chinese policy texts. Locations refer to the source documents used during coding. Each thematic segment was assigned to one primary policy-instrument node according to its dominant regulatory intention and primary governance function.
